# The No-U-Turn sampler and its mixture-modeling revision for the 4-parameter normal ogive model

**DOI:** 10.3389/fpsyg.2026.1746754

**Published:** 2026-03-31

**Authors:** Shaoyang Guo, Qian Sun, Xiaoyu Li

**Affiliations:** 1 School of Education and Intelligent Education Research Center, Yangzhou University, Yangzhou, China; 2 School of Education, Suzhou University of Science and Technology, Suzhou, China

**Keywords:** 4-parameter normal ogive model, bayesian estimation, Hamilton Monte Carlo, item response theory, revised No-U-Turn sampler

## Abstract

**Introduction:**

The No-U-Turn Sampler (NUTS), widely applied in psychometrics via the Stan platform, lacks algorithm-level systematic introduction for item response theory (IRT) models and tailored optimizations for specific models. This study systematically explicates the NUTS algorithm for the 4-parameter normal ogive (4PNO) model and proposes a Mixture-Modeling NUT sampler (MMNUTS) to enhance its estimation.

**Methods:**

A simulation study that considers both educational and psychological scenarios with an empirical example from a behavioral scale was designed to examine the feasibility of the proposed MMNUTS. A recoverability comparison is performed among the NUT sampler in Stan, MMNUTS, and the Gibbs-within-Gibbs sampler in the R package fourPNO.

**Results:**

All samplers achieved satisfactory convergence, with estimation accuracy improved at larger sample sizes. MMNUTS showed significant superiority in running time and upper asymptote parameter recovery in the psychological scenario with less informative priors and 5,000 samples. The 4PNO model yielded optimal fit in the empirical analysis, with MMNUTS demonstrating faster convergence and higher computational efficiency than the other two samplers.

**Discussion:**

The proposed MMNUTS maintains robust estimation accuracy while improving computational efficiency for the 4PNO model, providing a foundational reference for developing tailored sampling algorithms for complex psychometric models. The MATLAB codes for MMNUTS are presented in the appendix.

## 1 Introduction

Since the foundation of modern psychometrics, how to obtain more accurate parameters has been an inevitable issue with the development of psychological or educational models (Lord and Novick, 1968; Rasch, 1960). During the early phases of scholarly investigation, the family of maximum likelihood estimation (MLE) made great contributions to solving the issue of parameter estimation, and it remains a valuable and widely used approach in psychometric research to this day (Bock and Aitkin, 1981; Bock and Lieberman, 1970; Lord, 1980). However, the increasing complexity of modern measurement models poses great challenges for MLE, as it requires a relatively large sample

size to yield stable and accurate estimates. In response to these challenges—particularly in scenarios with small, medium, or even large but complex samples—psychometricians introduced Bayesian priors into the estimation process (Mislevy, 1986; Swaminathan and Gifford, 1986), eventually developing the fully Bayesian Markov Chain Monte Carlo (MCMC) method with the Metropolis-Hastings sampler (Patz and Junker, 1999a,b) as a complementary and powerful alternative.

MCMC is a method with relatively broad applicability and high flexibility in modern statistical inference. Taking the Metropolis-Hastings sampler as an example, its core logic lies in constructing a Markov chain whose stationary distribution matches the target distribution to be sampled (Metropolis et al., 1953). Starting from an arbitrary initial state in the sample space, a new candidate state is proposed at each iteraction according to a predefined transition rule. A probabilistic acceptance-rejection criterion is then used to determine whether to adopt the candidate state or retain the current one. As the number of iterations increases, the chain converges to its stationary distribution, from which valid parameter estimates can be derived. Owing to this straightforward algorithmic logic, together with its robust performance and ease of implementation—facilitated by the BUGS software family—MCMC has become a general solution for parameter estimation, particularly for newly proposed or complex measurement models (Béguin and Glas, 2001; Patz and Junker, 1999b). Despite its widespread adoption, a major challenge of MCMC is its potentially low sampling efficiency, which can result in unacceptably long estimation runtimes in some cases (Luke, 1994), especially for complex item response models (IRT) with large sample sizes and limited prior information (Fu et al., 2021; Zheng et al., 2021). To address this limitation, researchers have continuously strived to develop more efficient sampling algorithms, including the Gibbs sampler (Geman and Geman, 1984), the Slice sampler (Persi et al., 2000), the Hamiltonian Monte Carlo method (Neal, 2011), and the No-U-Turn (NUT) sampler (Hoffman and Gelman, 2014).

After the continuous efforts of the aforementioned studies, the NUT sampler is currently the most effective solution for estimating the majority of psychometric models (Papaspiliopoulos et al., 2020). It draws on the idea of Hamilton dynamics to repeatedly use the gradient of likelihood function to explore in multiple sampling directions, thus greatly improving sampling efficiency (Neal, 2011). It should also be clarified that the core idea of using Hamiltonian dynamics is inherent to the Hamiltonian Monte Carlo (HMC) approach (Neal, 2011); the NUT sampler, as an optimized extension of HMC, mainly plays a role in simplifying the computation and guiding the search. Moreover, Carpenter et al. (2017) develops a general Bayesian estimation implementation tool Stan based on the NUT sampler, and it also has been formally introduced for psychometrics by Luo and Jiao (2017), although the basic IRT models (one/two parameter logistic IRT model) were already provided in the Stan user manual. Until now, Stan has been widely used for parameter estimation in both item response theory models (Ames and Au, 2018; Bürkner, 2021; Scharl and Gnambs, 2019; Xue and Chen, 2023) and cognitive diagnosis models (Jiang and Carter, 2019; Tan et al., 2023).

Unfortunately, although Stan greatly alleviated the efficiency problems of parameter estimation, it also reduced researchers' enthusiasm to further improve the NUT sampler, especially for developing algorithms tailored to specific psychometric models. In literature, no researchers have systematically introduced how to utilize the NUT sampler for psychometric models from an algorithmic perspective, and there are even no corresponding algorithmic revisions for specific models. The lack of literature regarding the NUT sampler at the algorithmic level is not conducive to further improving the estimation in the field of psychometrics, which also motivated this study to offer a systematical introduction and an exploratory improvement for the NUT sampler given a specific model from an algorithmic perspective.

Recently, another interesting topic concerning the estimation issue is how to obtain stable estimates of IRT models with asymptote parameters. Specifically, a series of mixture-modeling-based Bayesian EM approaches were developed for Birnbaum (1968)'s 3PLM (Guo and Zheng, 2019; Guo et al., 2020), Barton and Lord (1981)'s 4PLM (Meng et al., 2019; Zheng et al., 2021), and San Martín et al. (2006) one-parameter ability-based guessing (1PL-AG) model (Guo et al., 2021); and several Gibbs-based samplers were proposed for the uni- or multi-dimensional 4-parameter IRT models (Culpepper, 2015, 2017; Fu et al., 2021; Zhang et al., 2020). These efforts not only make the mixture-modeling-based Bayesian EM algorithms quickly obtain Bayesian estimates that are comparable to those from MCMC but also alleviate the time-consuming issue of MCMC. Furthermore, this study was also inspired by the above efforts to attempt to combine the mixture-modeling approaches and the NUT sampler, and then proposed a mixture-modeling NUT sampler (MMNUTS) to further improve estimating accuracy and save running time. Moreover, among models containing asymptote parameters, the 4-parameter normal Ogive (4PNO) model (with both upper and lower asymptote parameters) is the most representative and can be easily extended to models with only one asymptote parameter. In addition, existing research has focused primarily on the logistic model, with the normal ogive model receiving relatively less attention. Accordingly, this study uses the 4PNO model as an illustrative example for the subsequent discussion.

Following is the remainder of this manuscript: First, an introduction of the 4PNO model was reported to describe its item response function, applications, and implementations. Second, the NUT sampler for the IRT models was systematically introduced from an algorithmic perspective using the 4PNO model as an example. Third, the MMNUT sampler which imposes the mixture-modeling revision to the NUT sampler for the 4PNO model was deliberated. Fourth, several item parameter recovery metrics, including bias, root mean square error (RMSE), and averaged posterior standard deviation (PSD), were compared between the MMNUT sampler, the original NUT sampler in R package rstan (Stan Development Team, 2022), and the Gibbs-within-Gibbs sampler in R package fourPNO (Culpepper, 2015). Fifth, the superiority of the MMNUTS within the 4PNO framework is demonstrated through an empirical example, by comparing Gelman–Rubin potential scale reduction factors, point estimates, associated standard errors, and computation time. Lastly, the major findings and future directions were discussed.

## 2 The No-U-Turn sampler for item response theory models

### 2.1 The 4-parameter normal ogive model

The item response function of the 4PNO model given a correct response *y*_*ij*_ = 1 in Equations (1, 2) is defined by Culpepper (2015) as


$$\begin{array}{c} P\left(y_{ij}=1|\theta_{i},\varpi_{j}\right)\equiv P_{ij}=g_{j}+\left(1-s_{j}-g_{j}\right)P_{ij}^{*}, \end{array}$$ (1)

with,


$$\begin{array}{c} P_{ij}^{*}=\Phi \left(\alpha_{j}\theta_{i}-\beta_{j}\right), \end{array}$$ (2)

where, Φ(·) is the cumulative distribution function of the standard normal distribution; *y*_*ij*_∈*Y* represents the response of examinee *i*(*i* = 1, 2, ..., *I*) on item *j*(*j* = 1, 2, ..., *J*); $\varpi_{j}={\left[\alpha_{j},\beta_{j},g_{j},s_{j}\right]}^{T}\in W$ collects the item slope α_*j*_, threshold β_*j*_, lower asymptote *g*_*j*_, and upper asymptote *s*_*j*_ parameters for item *j*; θ_*i*_∈**θ** is the ability parameter for subject *i*.

In recent years, the significance of asymptote parameters has drawn more attention in both educational and psychological assessments (Kern and Culpepper, 2020; Waller and Feuerstahler, 2017). In educational scenarios, the *g*_*j*_ and *s*_*j*_ parameters were traditionally regarded as the guessing and slipping parameters, respectively. Furthermore, several studies have shown that the *s*_*j*_ parameter is particularly valuable for low-stakes and large-scale assessments, which can be used to recover the mistakes caused by the lack of motivation of high-performing students (Culpepper, 2017; Green, 2011). In psychological scenarios, the *g*_*j*_ and *s*_*j*_ parameters can be respectively interpreted as the effect of malingering responses and the effect of social desirability bias, especially for those sensitive tests (Guo et al., 2023; Waller and Reise, 2010).

To obtain the estimates of the 4PNO model, Culpepper (2015) proposed a Gibbs-within-Gibbs sampler and then developed a R package four PNO, and Meng and Xu (2022) proposed a mixed stochastic approximation EM (MSAEM) algorithm to compute the marginalized maximum a posteriori estimator. Based on their efforts, this study aims to further examine the feasibility of the NUT sampler and its mixture-modeling revision for the 4PNO model.

### 2.2 The No-U-Turn sampler for the 4PNO model

Using the 4-parameter normal ogive (4PNO) model as an example, this section will illustrate how the NUT sampler operates on IRT models. First, the likelihood function of 4PNO model can be defined in Equation (3) as


$$\begin{array}{c} L\left(\theta ,W|Y\right)=\overset{I}{\underset{i=1}{\prod }}\overset{J}{\underset{j=1}{\prod }}\left[P_{ij}^{y_{ij}}{\left(1-P_{ij}\right)}^{1-y_{ij}}\right]. \end{array}$$ (3)

Then, let *g*(•) be the prior probability density function for item or ability parameters, then the log-likelihood function for the parameters of item *j* or examinee *i* after incorporating Bayesian prior should be


$$\begin{array}{c} \begin{array}{c} l_{\varpi_{j}}=\ln\;\overset{I}{\underset{i=1}{\prod }}\left[P_{ij}^{y_{ij}}{\left(1-P_{ij}\right)}^{1-y_{ij}}\right]\left[g\left(\varpi_{j}\right)\right]\\ l_{\theta_{i}}=\ln\;\overset{J}{\underset{j=1}{\prod }}\left[P_{ij}^{y_{ij}}{\left(1-P_{ij}\right)}^{1-y_{ij}}\right]\left[g\left(\theta_{i}\right)\right] \end{array}, \end{array}$$ (4)

Furthermore, the gradients of the item or ability parameters in Equation (4) can be expressed as


$$\begin{array}{c} l_{\varpi_{j}}^{\prime }=\frac{\partial l_{\varpi_{j}}}{\partial \varpi_{j}}=\frac{\partial \ln g\left(\varpi_{j}\right)}{\partial \varpi_{j}}+\sum_{i=1}^{I}\left[\frac{y_{ij}-P_{ij}}{P_{ij}\left(1-P_{ij}\right)}\frac{\partial P_{ij}}{\partial \varpi_{j}}\right]\\ l_{\theta_{i}}^{\prime }=\frac{\partial l_{\theta_{i}}}{\partial \theta_{i}}=\frac{\partial \ln g\left(\theta_{i}\right)}{\partial \theta_{i}}+\sum_{j=1}^{J}\left[\frac{y_{ij}-P_{ij}}{P_{ij}\left(1-P_{ij}\right)}\frac{\partial P_{ij}}{\partial \theta_{i}}\right]. \end{array}$$ (5)

**Figure 1:**
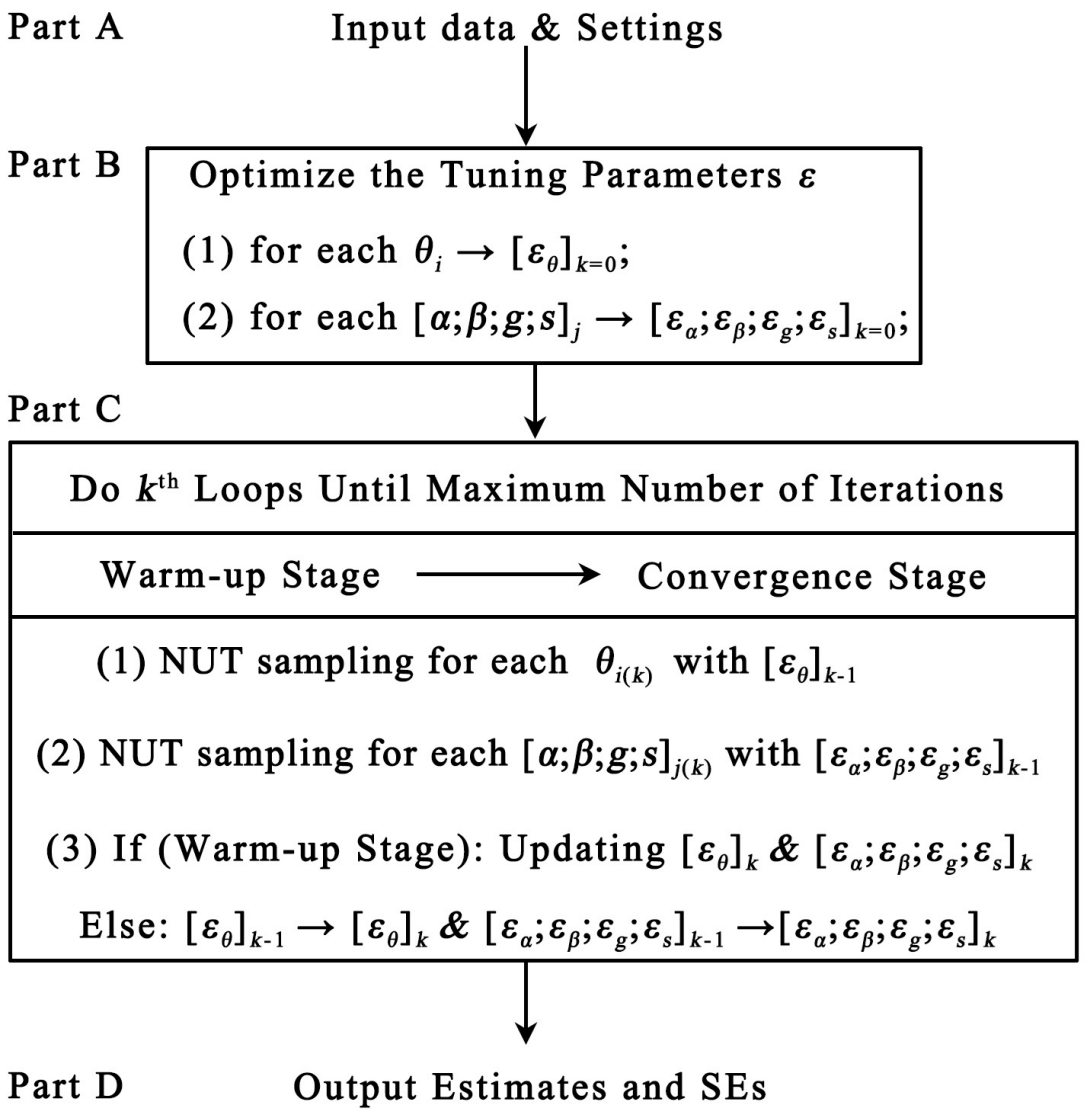
The flow chart of the No-U-Turn sampler for the 4PNO model.

Compared with the MH or Gibbs sampler, the NUT sampler is far more complicated. To facilitate understanding, this section will first give a macro estimating framework for the NUT sampler, and then discuss the technical details for each part of the framework. Basically, the NUT sampler estimates model parameters through Hamiltonian dynamics-augmented MCMC sampling, where tuning parameters (step size and trajectory length) are critical to tune the trade-off between proposal acceptance rate and sampling speed. Compared with the original HMC algorithm, a significant improvement of the NUT sampler is that it can automatically find the optimal tuning parameters, thereby enabling it to perform tasks more efficiency and robust. By optimizing these parameters, the NUT sampler generates guided parameter candidates that converge to the target posterior distribution, ensuring reliable and accurate parameter estimates for high-dimensional statistical models. As illustrated in Figure 1, the NUT sampler for the 4PNO model may be separated into four parts, each of which will be described in detail below. In addition, the corresponding MATLAB codes were also reported in Appendix A for reference.

**Part A: Input data & settings**. To produce the Bayesian estimates of the 4PNO model, the NUT sampler requires several preconditions, such as the response matrix *Y*, the initial values [ϖ_0_, θ_0_] and corresponding priors *g*(•) for item and ability parameters, the number of maximum iterations (*k* = 1, 2, ..., *K*). In addition, the NUTS sampler necessitates the initialization of certain constant parameters to facilitate computations. Given that these values require no manual adjustment in most scenarios and to minimize redundant statistical notation, this study directly incorporates these fixed constants into subsequent calculations, following the recommendation of Hoffman and Gelman (2014). For instance, the constant 0 is used in the expression $H_{\varpi_{0}}=0_{4}^{T}$ in Step B5 of Part B of the following text, the constant 10 is used in the expression $\eta_{a}={\left(k+10\right)}^{-1}$ in Step C1 of Part C below. For further details, interested readers are referred to the work of Algorithm 6 from Hoffman and Gelman (2014) for details. Once the data and settings have been inputted, the NUT sampler will move to Part B.

**Part B: Optimize the tuning parameter** **ε**. The tuning parameter **ε** is crucial for the estimation process of the NUT sampler, as it determines how much the estimating parameters should move in each iteration. To guarantee the accuracy and convergence of the estimation, the NUT sampler must find an initial reasonable **ε** for each item or ability parameter. Therefore, Part B was designed to specifically demonstrate how to obtain the optimized **ε** for each parameter.

The item response theory (IRT) model involves two key parameter types: examinee ability parameters and item parameters. In practice, the Markov chain Monte Carlo (MCMC) algorithm adopts a joint estimation approach for these parameters: it first updates ability parameters by treating item parameters as known, then iteratively refines item parameters using the updated ability estimates, and repeats this cycle until convergence. Given the high similarity between the optimization and sampling procedures for item and ability parameters, the subsequent sections focus exclusively on illustrating the process of updating item parameters under the assumption that ability parameters are fixed and known. Once all of the optimized tuning parameters are obtained, the NUT sampler will move to Part C. The tuning parameters **ε** for item *j* can be optimized via following steps:

**Step B1:** Obtain $\left[l_{\varpi_{0}},l_{\varpi_{0}}^{\prime }\right]$ based on initial values [ϖ_0_, θ_0_] from Equations (4, 5).

**Step B2:** Let $\varepsilon_{\varpi_{0}}=1_{4}^{T}={\left[1,1,1,1\right]}^{T}$ where *T* represents the transpose symbol, and then randomly sample *r*_ϖ_0__ from $MVN\left(0_{4}^{T},I_{4}\right)$, where *I*_4_ is a 4-dimensional identity matrix.

**Step B3:** Obtain $\left[\varpi_{1},r_{\varpi_{1}},l_{\varpi_{1}}^{\prime },l_{\varpi_{1}}\right]$ from *LeapFrog*(ϖ_0_, ε_ϖ_0__, *r*_ϖ_0__), and Equations (4, 5), where the function *LeapFrog*(ϖ_0_, ε_ϖ_0__, *r*_ϖ_0__) can be defined as


$$\begin{array}{c} r_{\varpi_{1}}^{*}=r_{\varpi_{0}}+\frac{\varepsilon_{\varpi_{0}}}{2}\circ l_{\varpi_{0}}^{\prime }\Rightarrow \varpi_{1}=\varpi_{0}+\varepsilon_{\varpi_{0}}\circ r_{\varpi_{1}}^{*}\\ \Rightarrow r_{\varpi_{1}}=r_{\varpi_{1}}^{*}+\frac{\varepsilon_{\varpi_{0}}}{2}\circ l_{\varpi_{1}}^{\prime }, \end{array}$$

where ° represents Hadamard Product (the element-wise multiplication of their corresponding components). It is worth noting that Step B3 actually implements a LeapFrog process of HMC. Specifically, the Leapfrog integrator discretizes Hamiltonian dynamics via a 3-step process: half-step momentum update, full-step position update, and a final half-step momentum update. This staggered small-step scheme accurately approximates continuous system trajectories while conserving energy. Moreover, in the Leapfrog step, the momentum term *r* guides movement through the parameter space and corresponds to the particle's momentum. Endowing the sampler with inertia, *r* enables NUT sampler to move in smooth trajectories across the parameter space instead of the tiny, random steps of traditional MCMC, allowing the algorithm to jump over local “dead ends” and quickly reach high-probability regions for greater efficiency. During LeapFrog process, *r* is updated in small steps alongside the target distribution's gradient. Critically, *r* is randomly reset at the start of each iteration to ensure sampling fairness and eventual convergence to the true target distribution, while using momentum to explore far faster than standard random walk methods.

**Step B4:** Calculate the optimal criteria δ = [Ω^ρ^>2^−ρ^], where [•] is Iverson's bracket, ρ = 2[Ω>0.5]−1 represents the determination of the direction of transition with the value of 1 or -1, Ω = exp(ξ_1_−ξ_0_), $\xi_{1}=l\left(y_{j}|\omega_{1},\theta \right)-\frac{1}{2}r_{\varpi_{1}}^{T}r_{\varpi_{1}}$, $\xi_{0}=l\left(y_{j}|\omega_{0},\theta \right)-\frac{1}{2}r_{\varpi_{0}}^{T}r_{\varpi_{0}}$, and ξ_1_ and ξ_0_ are the weighted posterior probabilities given the estimates of ω_1_ and ω_0_, respectively.

**Step B5:** If δ = 1, then let $\varepsilon_{\varpi_{0}}\leftarrow 2^{\rho }\varepsilon_{\varpi_{0}}$ and do loops from Step B3 to B5; otherwise, return ε_ϖ_0__, and set ι_ϖ_0__ = ln (10ε_ϖ_0__), $\varepsilon_{{\bar{\omega }}_{0}}^{\prime }=1_{4}^{T}$, and $H_{\varpi_{0}}=0_{4}^{T}$, where ι_ϖ_0__ and *H*_ϖ_0__ were procedural parameters for program and calculation.

**Part C: Do loops until the maximum number of MCMC iterations**. Part C is the core sampling algorithm of the NUT sampler, which consists of two stages of *K*(*k* = 1, 2, ..., *K*) MCMC iterations: The warm-up stage for burn-in and the convergence stage for generating the posterior distribution. Specifically, the sampling procedures of the two stages are almost the same, the only difference is that the warm-up stage needs to further update **ε**, while the convergence stage does not. In each MCMC iteration, the so-called function *BuildTree(**)* is the most complicated process. Intuitively speaking, it is just like a binary tree with *M* branches. In every *t*∈[1, 2, ..., *M*] growth (or multisampling), a direction will be randomly selected from the positive (+) and negative (-) ways for exploration and then iterated to *M* growth on this basis. Thus, the Bayesian sampling for the *k*^*th*^ iteration can be yielded via the following steps:

**Step C1:** Update $l_{\varpi_{0}}^{\prime }$ and ξ_0_ (see Step B3 and B4) by resampling *r*_ϖ_0__ from $MVN\left(0_{4}^{T},I_{4}\right)$. Then, a reference value should be randomly sampled to assist in determining whether the new value should be updated during subsequent parameter updates. It also should be noted that in the original NUT sampler by Hoffman and Gelman (2014), this reference value was designed to verify whether the updated $e^{\xi_{1}}$ is sufficiently probable to be larger than current $e^{\xi_{0}}$, and should thus be randomly sampled from *U*[0, exp(ξ_0_)]. Since exponentiation is computationally expensive, this study instead compares their logarithmic forms in practice. To implement this approach, referred to NUTS-matlab project (https://github.com/aki-nishimura/NUTS-matlab) developed by Hoffman's research team and calculated *u*_0_ = ξ_0_−*x*, where *x* is randomly sampled from the Exponential distribution *Exp*(1). This allows the algorithm to directly compare ξ_1_ and *u*_0_, thereby reducing computational costs. Third, let $\varpi_{0}^{-}$, $\varpi_{0}^{+}$ and ϖ_1_ be initialized with the value ϖ_0_; $r_{\varpi_{0}^{-}}$ and $r_{\varpi_{0}^{+}}$ be initialized with the value *r*_ϖ_0__; $l_{\varpi_{0}^{-}}$ and $l_{\varpi_{0}^{+}}$ be initialized with the value $l_{\varpi_{0}}^{\prime }$. Finally, set *t* = 0, *n*_*a*_ = 1, $\eta_{a}={\left(k+10\right)}^{-1}$, and $\eta_{b}=k^{-0.75}$, where *n*_*a*_, η_*a*_, and η_*b*_ were procedural parameters for program and calculation.

**Step C2:** Randomly choose a positive or negative direction from *dir*∈[−1, +1], and then update parameters via the *BuildTree()* function in Box 1. More specifically, if *dir* = −1, set $\left(\varpi_{0}^{*}l_{\varpi_{0}^{*}}^{\prime }r_{\varpi_{0}^{*}}\xi_{0}^{*}u_{0}^{*}t^{*}\right)\leftarrow \left(\varpi_{0}^{-},l_{\varpi_{0}^{-}},r_{\varpi_{0}^{-}},\xi_{0},u_{0},t\right)$ to return ϖ_1_, $\varpi_{1}^{-}$, $r_{\varpi_{1}^{-}}$, $l_{\varpi_{1}^{-}}^{\prime }$, *n*_*b*_, υ_*a*_, λ_*a*_ and λ_*b*_; otherwise, set $\left(\varpi_{0}^{*}l_{\varpi_{0}^{*}}^{\prime }r_{\varpi_{0}^{*}}\xi_{0}^{*}u_{0}^{*}t^{*}\right)\leftarrow \left(\varpi_{0}^{+},l_{\varpi_{0}^{+}}^{\prime },r_{\varpi_{0}^{+}},\xi_{0},u_{0},t\right)$ to return ϖ_1_, $\varpi_{1}^{+}$, $r_{\varpi_{1}^{+}}$, $l_{\varpi_{1}^{+}}^{\prime }$, *n*_*b*_, υ_*a*_, λ_*a*_ and λ_*b*_, where *n*_*b*_, υ_*a*_, λ_*a*_, and λ_*b*_ were procedural parameters for program and calculation.

Box 1 **The pseudo-codes for function *BuildTree()*.**

Function suildTreel (*dir*, $\varpi_{0}^{*}$ $l_{\varpi_{0}^{*}}^{\prime }$, $r_{\varpi_{0}^{*}}$, $\xi_{0}^{*}$, $u_{0}^{*},t^{*}$, ε_ϖ_0__ θ, **y**_*p*_, *M*)

% *dir* is a number indicating the sampling direction valued from [−1, 1].

% $\varpi_{0}^{*}$ Is is a 4 × 1 vector containing the current item estimates of the 4PNO model for Item, *j*.

% $l_{\varpi_{0}^{*}}^{\prime }$ is a 4 × 1 vector containing the first derivatives given $\varpi_{0}^{*}$

% *r*_ϖ_0__ is a 4 × 1 vector randomly sampled from $MVN\left(0_{\text{s}}^{\text{T}},{\text{I}}_{4}\right)$ for LeapFrog(ϖ_0_, ε_ϖ_0__, *r*_ϖ_0__).

$\%\xi_{0}^{*}$ is a number collecting the weighted posterior probabilities given ω_0_ from Step B4.

% $u_{0}^{*}$ is a number for controlling loops obtained from S tep C 1.

% ε_ϖ_0__ is a 4 × 1 vector collecting the tuning parameters obtained by Step B5.

% t^*^ is a number indicating the number of current branches in the binary trees.

*%θ* is a 1 × 1 vector containing the ability parameters for all examinees.

% **y**_*j*_ is a *l*×1 vector containing the responses of Item *j*.

% *M* is a number indicating the max branches *M*(*t* = 0, 1, …, *M*) of the binary trees.

If t^*^ = 1 :

(1) Obtain $\left[\varpi_{1}^{*},r_{\varpi_{1}^{*}},l_{\varpi_{1}^{*}}^{\prime },l_{\varpi_{1}^{*}}\right]$ via (ϖ_0_, ε_ϖ_0__, *r*_ϖ_0__) process referring to Step B3;

(2) Calculate $\xi_{1}^{*}=l\left(y_{j}\text{|}\omega_{1}^{*}\text{,}\theta \right)-\frac{1}{2}r_{\varpi_{1}^{*}}^{T}r_{\varpi_{1}^{*}}$;, where *T* is the transpose symbol;

(3) Update $n_{b}=\left[u_{0}^{*}<\xi_{1}^{*}\right]$ $\upsilon_{a}=\left[\left(u_{0}^{*}-100\right)\geq \xi_{1}^{*}\right]$ $\lambda_{a}=\min\left(1,\exp\left(\overset{*}{\underset{1}{\xi }}-\xi_{0}^{*}\right)\right)$

(4) Set $\left(\varpi_{1},l_{\varpi_{1}}^{\prime },r_{\varpi_{1}}\right)\leftarrow \left(\varpi_{1}^{*}l_{\varpi_{1}^{*}}^{\prime }r_{\varpi_{1}^{*}}\right)$, $\left(\varpi_{1}^{-},l_{\varpi_{1}^{-}}^{\prime },r_{\varpi_{1}^{-}}\right)\leftarrow \left(\varpi_{1}^{*}l_{\varpi_{1}^{*}}^{\prime }r_{\varpi_{1}^{*}}\right)$,

$\left(\varpi_{1}^{+},l_{\varpi_{1}^{+}}^{\prime },r_{\varpi_{1}^{+}}\right)\leftarrow \left(\varpi_{1}^{*}l_{\varpi_{1}^{*}}^{\prime }r_{\varpi_{1}^{*}}\right)$ and λ_*s*_←1;

else:

(1) Let t^*^←(t^*^−1) and then call *BuildTree()* to obtain the updated parameters;

(2) If (*v*_*a*_ = 0) :

(a) If (∣ dir = −1) : Replace $\left(\varpi_{0}^{*},l_{\varpi_{0}^{*}}^{\prime },{\text{r}}_{\varpi_{0}^{*}}\right)$ with $\left(\varpi_{1}^{-},l_{\varpi_{1}^{-}}^{\prime },{\text{r}}_{\varpi_{1}^{-}}\right)$to call *BuildTree()* again to gain $\left(\omega_{2}^{*},l_{\omega_{2}^{*}}^{\prime },{\text{r}}_{\omega_{2}^{*}}\right),\;\left(n_{b}^{\prime },v_{a}^{\prime },\lambda_{a}^{\prime },\lambda_{b}^{\prime }\right)$, and update $\left(\omega_{1}^{-},l_{\omega_{1}^{-}}^{\prime },{\text{r}}_{\omega_{1}^{-}}\right)$;

Else (dir = 1): Replace $\left(\varpi_{0}^{*},l_{\omega_{0}^{*}}^{\prime },{\text{r}}_{\omega_{0}^{*}}\right)$ with $\left(\varpi_{1}^{+},l_{\omega_{1}^{+}}^{\prime },{\text{r}}_{\omega_{1}^{+}}\right)$to call *BuildTree()* again to gain $\left(\omega_{2}^{*},l_{\omega_{2}^{*}}^{\prime },{\text{r}}_{\omega_{2}^{*}}\right),\;\left(n_{b}^{\prime },v_{a}^{\prime },\lambda_{a}^{\prime },\lambda_{b}^{\prime }\right)$ and update $\left(\omega_{1}^{+},l_{\omega_{1}^{+}}^{\prime },{\text{r}}_{\omega_{1}^{+}}\right);$

(b) Let $v_{b}=\left[x<\left(n_{b}^{\prime }/\left(n_{b}+n_{b}^{\prime }\right)\right)\right]$, where *x* is randomly sampled from *U*(0, 1).

If (*v*_*b*_ = 1) : Update $\left(\varpi_{1}^{*},l_{w_{1}^{*}}^{\prime },{\text{r}}_{w_{1}^{*}}\right)\leftarrow \left(\varpi_{2}^{*},l_{w_{2}^{*}}^{\prime },{\text{r}}_{w_{2}^{*}}\right);$

(c) Update $n_{b}\leftarrow n_{b}+n_{b}^{\prime },\lambda_{a}\leftarrow \lambda_{a}+\lambda_{a}^{\prime },\lambda_{b}\leftarrow \lambda_{b}+\lambda_{b}^{\prime }$, and $v_{a}\leftarrow \left[\left[\varpi_{1}^{T}{\text{r}}_{w_{1}^{-}}<0\right]+\left[\varpi_{1}^{T}{\text{r}}_{w_{1}^{+}}<0\right]+\left[v_{a}+v_{b}\neq 0\right]\neq 0\right]$, where $\varpi_{1}^{\prime }=\varpi_{1}^{+}-\varpi_{1}^{-}$.

Return $\left(\omega_{1},l_{\omega_{1}}^{\prime },{\text{r}}_{\omega_{1}}\right),\;\left(\omega_{1}^{-},l_{\omega_{1}^{-}}^{\prime },{\text{r}}_{\omega_{1}^{-}}\right),\;\left(\omega_{1}^{+},l_{\omega_{1}^{+}}^{\prime },{\text{r}}_{\omega_{1}^{+}}\right),n_{b},v_{a},\lambda_{a}$, and λ_*b*_.

**Step C3:** Calculate procedural parameter υ_*b*_ = [*x*≥(*n*_*b*_/*n*_*a*_)], where *x* is randomly sampled from *U*(0, 1). Then, update *n*_*a*_←*n*_*a*_+*n*_*b*_ and *t*←*t*+1. If υ_*a*_ = 0 and υ_*b*_ = 0, then update ϖ_0_←ϖ_1_. Note: *n*_*a*_ and *t* have been initialized in Step C1.

**Step C4:** Calculate $\upsilon_{c}=\left[\left[\varpi_{1}^{\prime }r_{\varpi_{1}^{-}}<0\right]+\left[\varpi_{1}^{\prime }r_{\varpi_{1}^{+}}<0\right]\neq 0\right]$ , where $\varpi_{1}^{\prime }\;=\varpi_{1}^{+}-\varpi_{1}^{-}$. If υ_*a*_ = 0 and υ_*c*_ = 0, then do loops from Step C1 to C4; otherwise, return ϖ_0_ and λ_*c*_ = λ_*a*_/λ_*b*_, where υ_*c*_, and λ_*c*_ were procedural parameters for program and calculation.

**Step C5:** If ***k* ≤ 0.5*K*** (a half of sampling were selected as the warm-up stage), then update *H*_ϖ_0__←(1−η_*a*_)*H*_ϖ_0__+η_*a*_(0.8−λ_*c*_), $\varepsilon_{\varpi_{0}}\leftarrow \exp\left(\iota_{\varpi_{0}}-\left(\sqrt{k}/0.05\right)H_{\varpi_{0}}\right)$, and $\varepsilon_{\varpi_{0}}^{\prime }\leftarrow \exp\left(\left(1-\eta_{b}\right)\ln\left(\varepsilon_{\varpi_{0}}^{\prime }\right)+\eta_{b}\ln\left(\varepsilon_{\varpi_{0}}\right)\right)$; otherwise (convergence stage), remain ε_ϖ_0__ unchanged. Note: ι_ϖ_0__ and *H*_ϖ_0__ have been initialized in Step B5.

**Part D: Output estimates and standard errors**. Collecting the Bayesian samplings from the convergence stage of Part D to build a posterior distribution for each parameter, and then the Bayesian estimates and standard errors (SEs) can be obtained by computing the mean and standard deviations of the corresponding posterior distribution.

## 3 The mixture-modeling No-U-Turn sampler for the 4PNO model

### 3.1 Mixture-modeling reformulation

The mixture-modeling reformulation has been widely used in the Bayesian estimation for the IRT models with asymptotes (Culpepper, 2015; Guo et al., 2023, 2021; Zheng et al., 2021). Following Béguin and Glas (2001), a latent dichotomous variable $z_{ij}\sim \;Bernoulli\left(P_{ij}^{*}\right)$ in Equation (6) has been introduced by Culpepper (2015) to provide a conditional probability of a correct response given *z*_*ij*_∈**Z** as


$$\begin{array}{c} y_{ij}|z_{ij},\varpi_{j},\theta_{i}\sim \{\begin{array}{cc} Bernoulli\left(1-s_{j}\right), & z_{ij}=1\\ Bernoulli\left(g_{j}\right), & z_{ij}=0 \end{array} \end{array}$$ (6)

where *z*_*ij*_ = 1 means the examinee *i* knows the answer of item *j*, so the unslipping probability 1−*s*_*j*_ will determine whether he can answer correctly; and *z*_*ij*_ = 0 means the examinee *i* does not know the answer of item *j*, so the guessing probability *g*_*j*_ will limit the correct response. Instead of using the conditional probability in the Gibbs-within-Gibbs sampler (Culpepper, 2015), the joint probability of (*y*_*ij*_, *z*_*ij*_) in Equation (7) was used in this study as


$$\begin{array}{c} P\left(y_{ij},z_{ij}|\varpi_{j},\theta_{i}\right)\sim \{\begin{array}{cc} \left(1-s_{j}\right)P_{ij}^{*}, & y_{ij}=1\text{ \& }z_{ij}=1\\ s_{j}P_{ij}^{*}, & y_{ij}=0\text{ \& }z_{ij}=1\\ g_{j}\left(1-P_{ij}^{*}\right), & y_{ij}=1\text{ \& }z_{ij}=0\\ \left(1-g_{j}\right)\left(1-P_{ij}^{*}\right), & y_{ij}=0\text{ \& }z_{ij}=0 \end{array}. \end{array}$$ (7)

Thus, the likelihood function of (*y*_*ij*_, *z*_*ij*_) in Equation (8) and the corresponding log-likelihood function in Equation (9) should be


$$\begin{array}{c} L(\theta ,W|Y,Z)=\prod_{i=1}^{I}\prod_{j=1}^{J}\left[\begin{array}{c} {\left[\left(1-s_{j}\right)P_{ij}^{*}\right]}^{y_{ij}z_{ij}}\times {\left[g_{j}\left(1-P_{ij}^{*}\right)\right]}^{y_{ij}\left(1-z_{ij}\right)}\\ \times {\left[s_{j}P_{ij}^{*}\right]}^{\left(1-y_{ij}\right)z_{ij}}\times {\left[\left(1-g_{j}\right)\left(1-P_{ij}^{*}\right)\right]}^{\left(1-y_{ij}\right)\left(1-z_{ij}\right)} \end{array}\right], \end{array}$$ (8)

and


$$\begin{array}{c} \ln L(\theta ,W|Y,Z)=\sum_{i=1}^{I}\sum_{j=1}^{J}\left[\begin{array}{l} z_{ij}\ln P_{ij}^{*}+\left(1-z_{ij}\right)\ln\left(1-P_{ij}^{*}\right)\\ +y_{ij}\left(1-z_{ij}\right)\ln g_{j}+\left(1-y_{ij}\right)\left(1-z_{ij}\right)\ln\left(1-g_{j}\right)\\ \left(1-y_{ij}\right)z_{ij}\ln s_{j}+y_{ij}z_{ij}\ln\left(1-s_{j}\right) \end{array}\right].. \end{array}$$ (9)

### 3.2 Revisions to the original NUT sampler

As for the mixture-modeling reformulated 4PNO model, suppose *z*_*ij*_ is known, then *L*(**θ**, **W** | **Y**, **Z**) will be exactly equal to *L*(**θ**, **W** | **Y**), so it is reasonable to maintain the traditional likelihood *L*(**θ**, **W** | **Y**) for finding the optimal estimates. However, compared with other MCMC samplers that only use the ratio of log-likelihood, one significant feature of the NUT sampler is the use of gradients. In this case, the unobservable parameter *z*_*ij*_ becomes a major obstacle for the NUT sampler to obtain the gradients from *L*(**θ**, **W** | **Y**).

Following Guo et al. (2023), this study first takes the expectation of the log-likelihood given *z*_*ij*_ to obtain *E*[ln *L*(**θ**, **W** | **Y**, **Z**)], and then uses the conditional expectation *E*(*z*_*ij*_|*y*_*ij*_, **θ**_*i*_, ϖ_*j*_) in Equation (10) to approximate *z*_*ij*_ to solve the expected log-likelihood. By Bayes rules,


$$\begin{array}{c} E\left(z_{ij}|y_{ij},\theta_{i},\varpi_{j}\right)\equiv z_{ij}^{\left(E\right)}=\frac{\left(1-s_{j}\right)P_{ij}^{*}}{P_{ij}}\times y_{ij}+\frac{s_{j}P_{ij}^{*}}{1-P_{ij}}\times \left(1-y_{ij}\right). \end{array}$$ (10)

To facilitate the robustness of the sampling (Mislevy, 1986), let $\alpha_{j}^{*}=\ln\;\alpha_{j}$, γ_*j*_ = ln(*g*_*j*_/(1−*g*_*j*_)), ς_*j*_ = ln(*s*_*j*_/(1−*s*_*j*_)), and ϕ(▪) be the density function of the normal distribution with prior mean μ and standard deviation σ, then the corresponding gradients for each type of parameters of the expected log-likelihood with Bayesian priors in Equations (11–15) can be deduced as (see Appendix B for detailed mathematical derivations)


$$\begin{array}{c} l_{\theta_{i}^{\prime }}=-\frac{\theta_{i}-\mu_{\theta_{i}}}{\sigma_{\theta_{i}}^{2}}+\sum_{j=1}^{J}\frac{z_{ij}^{\left(E\right)}-P_{ij}^{*}}{P_{ij}^{*}\left(1-P_{ij}^{*}\right)}e^{\alpha_{j}^{*}}\times \phi \left(e^{\alpha_{j}^{*}}\theta_{i}-\beta_{j}\right),, \end{array}$$ (11)

$$\begin{array}{c} l_{\alpha_{j}^{*}}^{\prime }=-\frac{\alpha_{j}^{*}-\mu_{\alpha_{j}^{*}}}{\sigma_{\alpha_{j}^{*}}^{2}}+\sum_{i=1}^{I}\frac{z_{ij}^{\left(E\right)}-P_{ij}^{*}}{P_{ij}^{*}\left(1-P_{ij}^{*}\right)}e^{\alpha_{j}^{*}}\theta_{i}\times \\ \phi \left(e^{\alpha_{j}^{*}}\theta_{i}-\beta_{j}\right), \end{array}$$ (12)

$$\begin{array}{c} l_{\beta_{j}}^{\prime }=-\frac{\beta_{j}-\mu_{\beta_{j}}}{\sigma_{\beta_{j}}^{2}}-\sum_{i=1}^{I}\frac{z_{ij}^{\left(\text{L}\right)}-P_{ij}^{*}}{P_{ij}^{*}\left(1-P_{ij}^{*}\right)}\times \phi \left(e^{\alpha_{j}^{*}}\theta_{i}-\beta_{j}\right), \end{array}$$ (13)

$$\begin{array}{c} l_{\gamma_{j}^{\prime }}^{\prime }=-\frac{\gamma_{j}-\mu_{\gamma_{j}}}{\sigma_{\gamma_{j}}^{2}}+\sum_{i=1}^{I}\left(y_{ij}-\mathrm{Invlogit}\left(\gamma_{j}\right)\right)\left(1-z_{ij}^{\left(E\right)}\right), \end{array}$$ (14)

$$\begin{array}{c} l_{\zeta_{j}^{\prime }}^{\prime }=-\frac{\varsigma_{j}-\mu_{\varsigma_{j}}}{\sigma_{\varsigma_{j}}^{2}}+\sum_{i=1}^{I}\left(1-y_{ij}-\mathrm{Invlogit}\left(\varsigma_{j}\right)\right)\times z_{ij}^{\left(E\right)}, \end{array}$$ (15)

where, Invlogit(*x*) = [1+exp(−*x*)]^−1^, and ϕ(·) is the probability density function of the standard normal distribution. By substituting the above gradients into Parts B & C of the NUT sampler, the Bayesian estimates can be yielded for the mixture-modeling reformulated 4PNO model.

## 4 Simulation studies

### 4.1 Research objectives

The simulation studies compared the item parameter recovery of the MMNUT sampler (MMNUTS) with that of the original NUT sampler in R package rstan (Stan Development Team, 2022) and the Gibbs-within-Gibbs sampler proposed by Culpepper (2015) in R package fourPNO. The MMNUTS algorithm is implemented in MATLAB R2023b, as MATLAB offers optimized LAPACK/BLAS linear algebra libraries and provides higher code readability by eliminating explicit compilation steps. To ensure numerical reliability, random seeds are fixed in MATLAB and R, rstan and fourPNO are configured per developer recommendations, and simulations of pre-researches confirm parameter estimates are consistent within numerical precision across software, with minor differences in implementation, numerical precision, and underlying libraries acknowledged but not the focus of this work. The recoverability of three samplers was evaluated by simulating various design features, including the location of item thresholds (educational vs. psychological situations) and sample sizes (2,500 vs. 5,000). The simulations are conducted in MATLAB R2023b, and the MATLAB scripts for MMNUTS and the simulated true item parameters are available in online Appendices A, C, respectively.

### 4.2 Design features and data generation

Simulating educational and psychopathological scenarios for the 4PNO model is essential due to the distinct psychometric features and estimation challenges of the two domains. According to Culpepper (2015), educational assessments have item parameters clustered at the center of the latent continuum, while psychopathology ones feature larger difficulty parameters that make estimating the upper asymptote far harder, and simulating both quantifies how these differences impact 4PNO parameter recovery accuracy. Additionally, the two domains interpret the 4PNO's asymptote parameters differently, *g*_*j*_and *s*_*j*_ stand for guessing/slipping in education and false endorsement/under-reporting in psychopathology, and dual simulations validate the model's ability to recover these substantively distinct parameters across contexts, thus confirming its generalizability. Both educational and psychological situations with two sample sizes (2,500 and 5,000) were simulated, and in both cases, the ability parameters θ_*i*_ were sampled from the standard normal distribution. Since the four-parameter model is particularly valuable for low-stakes educational assessments, the estimator should have the power to recover the widely distributed parameters in practice, especially for both asymptote parameters. Based on the Fu et al. (2021), Meng and Xu (2022) and Culpepper (2015), the item slope, thresholds, guessing, and slipping parameters for 20 items were generated from α_*j*_~ *U*(0.5, 3), β_*j*_~ *N*(0, 1), *g*_*j*_~ *U*(0.05, 0.35), and *s*_*j*_~ *U*(0.05, 0.35), respectively. Specifically, the mean, standard deviation (SD), minimum, and maximum values of simulated item discrimination, difficulty (β/α), guessing, and slipping parameters using traditional IRT metrics were (1.48, 0.67, 0.58, 2.82), (−0.04, 0.79, −2.10, 1.43), (0.19, 0.08, 0.08, 0.33), and (0.17, 0.06, 0.06, 0.26), respectively. Moreover, the Pearson correlation coefficient between *g* and *s* was −0.181.

For psychological tests, based on the 4PL calibration of adolescent self-esteem scale by Waller and Reise (2010), the item parameters of the 4PNO model reparametrized by Culpepper (2015) were used to simulate responses on 23 items. Specifically, the mean, SD, minimum, and maximum values of item discrimination, difficulty, guessing, and slipping parameters for psychological tests using traditional IRT metrics were (1.15, 0.33, 0.72, 1.95), (0.40, 0.64, −0.50, 1.56), (0.04, 0.05, 0.00, 0.24), and (0.26, 0.16, 0.05, 0.60), respectively. Moreover, the Pearson correlation coefficient between *g* and *s* was −0.308. Compared with the educational exams, the items on psychological tests have obviously larger difficulty parameters and social desirability bias (higher slipping but lower guessing), which may impair the recoverability since it is more challenging to recover the slipping parameters when the difficulty is larger (Culpepper, 2015).

### 4.3 Settings, priors, and item recovery criteria for three samplers

For each design feature, 100 replications with 4 chains were conducted to evaluate the recoverability of three samplers for item parameters. To avoid result discrepancies caused by varying initial values in the simulation study, identical initial values were used for each iteration. Following standard item response theory model conventions, initial values were generated as follows: (1) Item difficulty (correct rate), discrimination (item-total Pearson correlation), guessing (low-group correct rate), slipping (high-group error rate), and participant's average score rates were calculated via classical test theory. (2) These 0–1 bounded indicators were transformed to a normal scale using the inverse standard normal cumulative distribution function. (3) Initial item parameters were randomly sampled from a normal distribution (mean = transformed values, SD = 1) to ensure distinct initial values across chains. For both revised and original NUT samplers, 2,000 iterations (the first 1,000 iterations will be discarded as warm-up) were run to obtain the posterior distributions of item parameters, and the mean of the posterior distributions will be used to calculate the final estimates. Furthermore, all tuning parameters of both samplers adopted the default settings of the rstan package (see input variable “control” in function “stan” for details). For the Gibbs-within-Gibbs sampler, Culpepper (2015) suggested that at least 100,000 MCMC iterations (the first 50,000 iterations will be discarded as burn-in) were required for estimating the 4PNO model when thinning parameter equals 1, so 100,000 MCMC iterations were generated to obtain the Bayesian estimates in this study.

Considering the differences in algorithmic constraints and item parameterization, two types of priors were used for the MMNUTS/NUTS and the Gibbs-within-Gibbs sampler, respectively. In the educational situation, refer to Houts and Cai (2015), the normal-distributed priors were used for both the revised and original NUT sampler as ln α~ *N*(0, 0.5), $\beta \sim \;N\left(0,\sqrt{2}\right)$, (γ, ς)~ *N*(−1.39, 0.5) (Invlogit(−1.39)≈0.20); while the truncated Normal distribution and Beta distribution priors were used for the Gibbs-within-Gibbs sampler based on the suggestion of Culpepper (2015) and Zimowski et al. (2003) as: $\alpha \sim \;N\left(0,\sqrt{2}\right)I\left(\alpha >0\right)$, $\beta \sim \;N\left(0,\sqrt{2}\right)$ and (*g, s*)~ *Beta*(4, 16), where the practical expectation of α was about 1.13 due to its prior normal distribution being truncated at 0 and the hyper-parameter in *Beta* distribution is the reparameterization of (5, 17) in BILOG-MG (Zimowski et al., 2003) with the expectation of 0.20. In a more challenging psychological situation, to further check the robustness of the proposed sampler, relatively less informative priors were used for the asymptote parameters as (γ, ς)~ *N*(−1.61, 0.8) in both MMNUTS and NUTS and (*g, s*)~ *Beta*(1, 5) in the Gibbs-within-Gibbs sampler (Invlogit(−1.61)≈*E*(*Beta*(1, 5))≈0.17). In addition, the priors of ability parameters for all three samplers were θ~ *N*(0, 1).

The Gelman-Rubin potential scale reduction factors $\hat{R}<1.1$ from 4 chains (Gelman and Rubin, 1992) were used as convergence criteria. The root mean squared error (RMSE) and bias were used to assess the accuracy in recovering item parameters across the 100 replications where


$$\begin{array}{c} RMSE=\sqrt{\frac{{\sum_{r=1}^{R}\left({\hat{\varpi }}_{j}^{\left(r\right)}-\varpi_{j}\right)}^{2}}{R},}\;bias=\frac{\sum_{r=1}^{R}\left({\hat{\varpi }}_{j}^{\left(r\right)}-\varpi_{j}\right)}{R}, \end{array}$$

and *R* is the number of converged replications. Furthermore, the averaged posterior standard deviation (PSD) of item parameters was calculated to assess the variability of three samplers.

### 4.4 Results

**Figure 2:**
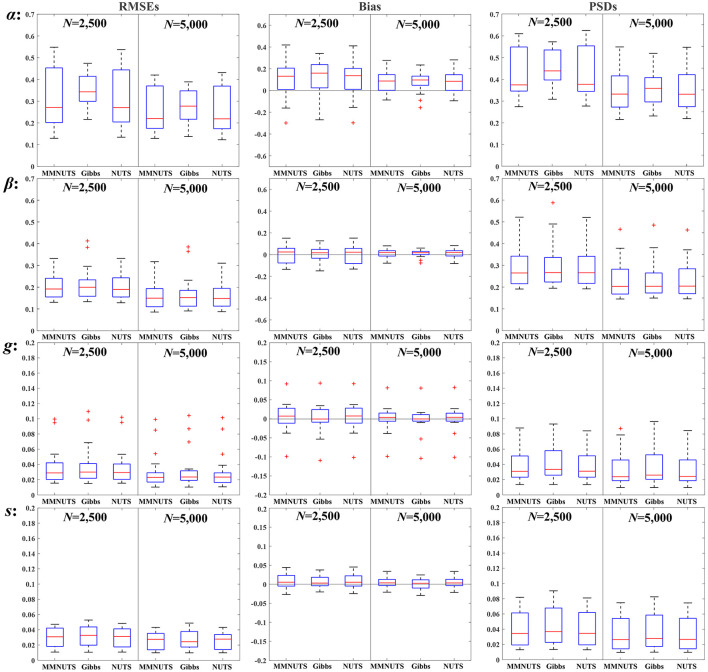
The RMSEs, bias, and PSDs for the educational scenario.

**Figure 3:**
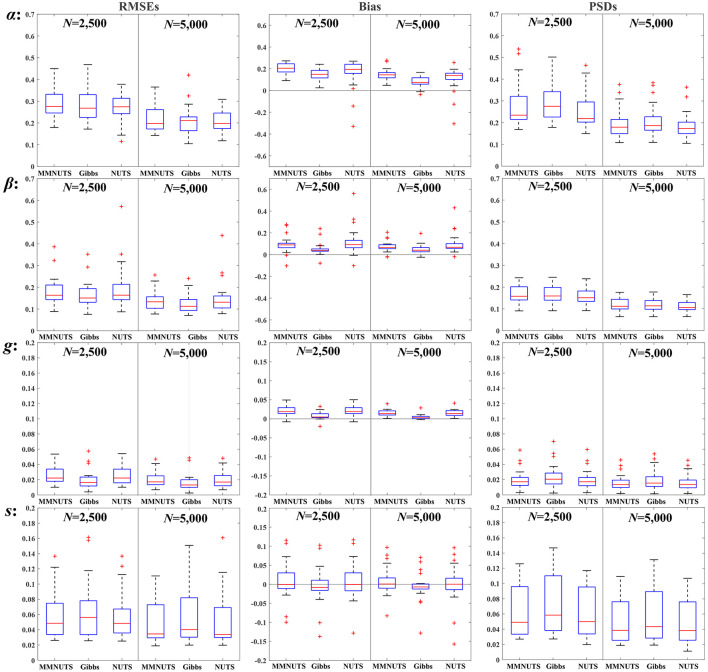
The RMSEs, bias, and PSDs for the psychological scenario.

Three samplers have achieved convergence in all replications, and the RMSEs, bias, and PSDs for educational and psychological scenarios were plotted in Figures 2, 3 (the complete results are also tabulated in Appendix D), respectively. It can be seen from the two figures that the increase of sample size from 2,500 to 5,000 led to substantial improvements in the accuracy of RMSEs and bias, as well as the variability of PSDs for all three samplers, which suggests that the 4PNO model is more suitable for sample sizes greater than 5,000. In general, the recoverability of all three samplers is comparable; nevertheless, their performance under educational and psychological contexts varies, especially for the slope and asymptote parameters.

Given the results in Figures 2, 3, the following findings can be observed about the item recovery of slope parameters: (1) Generally, the bias, RMSEs, and PSDs exhibited by MMNUTS and NUTS are very similar, yet they demonstrate discernible differences when contrasted with Gibbs-within-Gibbs sampler, especially for the RMSEs. (2) Averagely, MMNUTS and NUTS exhibit obviously smaller RMSEs compared to Gibbs-within-Gibbs in the educational domain, whereas their RMSEs were approximately comparable in the psychological field. (3) Compared to MMNUTS and NUTS, Gibbs-within-Gibbs shows a narrower range of variation of RMSEs in the educational scenario but a slightly broader range of RMSE variation in the psychological context. (4) The PSDs showed a consistent trend with RMSEs and bias.

The reason for these differences is probably related to the type and information of priors. In educational settings, due to the broad distribution of true values (α_edu_∈[0.58, 2.82]), the prior with a larger variance in the Gibbs-within-Gibbs method ($\alpha \sim \;N\left(0,\sqrt{2}\right)I\left(\alpha >0\right)$) allows for the easier recovery of aberrant values. Conversely, in psychological settings where the distribution of true values is narrower (α_psych_∈[0.72, 1.95]), the prior with a smaller variance used in MMNUTS and NUTS (ln α~ *N*(0, 0.5)) can provide more informative information to facilitate the recovery.

Regarding the asymptote parameters, estimating the lower and upper asymptotes presents distinct sets of challenges. For the educational settings (μ_*g*_ = 0.19 &* μ*_*s*_ = 0.17), the recovery of the guessing parameters is more difficult, and all three samplers yielded aberrant values in both bias and RMSEs, while the recovery of the slipping parameters is relatively feasible. However, for the psychological situation, although the recovery of guessing parameters appears satisfactory, the recovery of slipping parameters by the three samplers yields much worse RMSEs, bias, and PSDs than those from educational settings, particularly when considering a sample size of 2,500.

Despite this, both MMNUTS and NUTS still demonstrates certain advantages in recovering asymptote parameters when using relatively less informative priors. Specifically, in the educational setting with relatively informative priors ((γ, ς)~ *N*(−1.39, 0.5) and (*g, s*)~ *Beta*(4, 16)), the item recovery of guessing and slipping parameters by MMNUTS and NUTS are comparable to those from the Gibbs-within-Gibbs sampler; however, when the priors change to be less informative ((γ, ς)~ *N*(−1.61, 0.8) and (*g, s*)~ *Beta*(1, 5)) in the psychological settings, the RMSEs of slipping parameters by MMNUTS and NUTS are notably smaller than those from the Gibbs-within-Gibbs sampler. Moreover, there are even no aberrant RMSE values yielded from MMNUTS when the sample size increases to 5,000, indicates that MMNUTS can improve the accuracy of asymptote parameters by introducing the mixture-modeling approach.

Another interesting finding relates to the threshold parameters in the psychological scenario. It can be seen that there are several aberrant values of rstan appearing in both bias and RMSEs, while both fourPNO and MMNUTS remain stable. This observation may reflect potential differences between algorithms optimized for specific models and general Bayesian implementations, though such differences need to be interpreted with caution given the stochastic nature of the sampling process and the limited scope of the simulation. It is worth noting that Figure 3 (second row for the threshold β parameter) shows that the number of outliers is basically similar across all three routines, with rstan exhibiting one particularly prominent outlier. Additionally, the variability of rstan tends to decrease as the sample size increases. A similar pattern of outliers is also observable in the loading parameter estimates.

## 5 An empirical example

This example will demonstrate the usefulness of the 4PNO model with MMNUTS using a behavioral scale, as the simulations have presented the item recovery in educational and psychological settings. The datasets including the responses of 11,154 students to 12 Tobacco and Alcohol Use (TAU) items from the 2009-2010 Health Behavior in School-Aged Children (HBSC) research (Iannotti, 2013) were analyzed. The TAU items ask subjects “How frequently have you smoked cigarettes during the LAST 30 DAYS” or “Have you ever had so much alcohol that you were really drunk” (all 12 items can be found in Appendix E), and each item requires subjects to report their frequency of TAU using 2-7 incrementally categorical options (e.g., 0 = Never, 1 = Rarely, 2 = Every month, 3 = Every week, and 4 = Every day). Since all samples consist of students in grades 5 to 10, the distribution of TAU responses is extremely positively skewed, so the datasets can be dichotomized as “never → 0” and “else → 1”.

To demonstrate the superiority of the 4PNO model, the 2PNO, 3PNO, and 4PNO models were used to analyze the recoded binary datasets via MMNUTS, Gibbs-within-Gibbs sampler, and original NUTS. Specifically, all three samplers used the same setting as the first simulation study. Table 1 summarizes the model fits of five competitive models. Obviously, the AIC and BIC of the 2PNO model are smaller than those of the 3PNO model but bigger than those of the 4PNO model, suggesting the utility of the upper asymptote parameters.

**Table 1:** The model comparisons of empirical examples.

Model	Log-Likelihood	Parameters	AIC	BIC	*LR* tests to 4PNO		
					χ^2^	*df*	*P*
2PNO	−33685.31	24	67418.62	67594.29	402.68	24	*P* < 0.001
3PNO	−33777.54	36	67626.07	67890.57	587.14	12	*P* < 0.001
4PNO	−33483.97	48	67063.95	67415.28			

**Figure 4:**
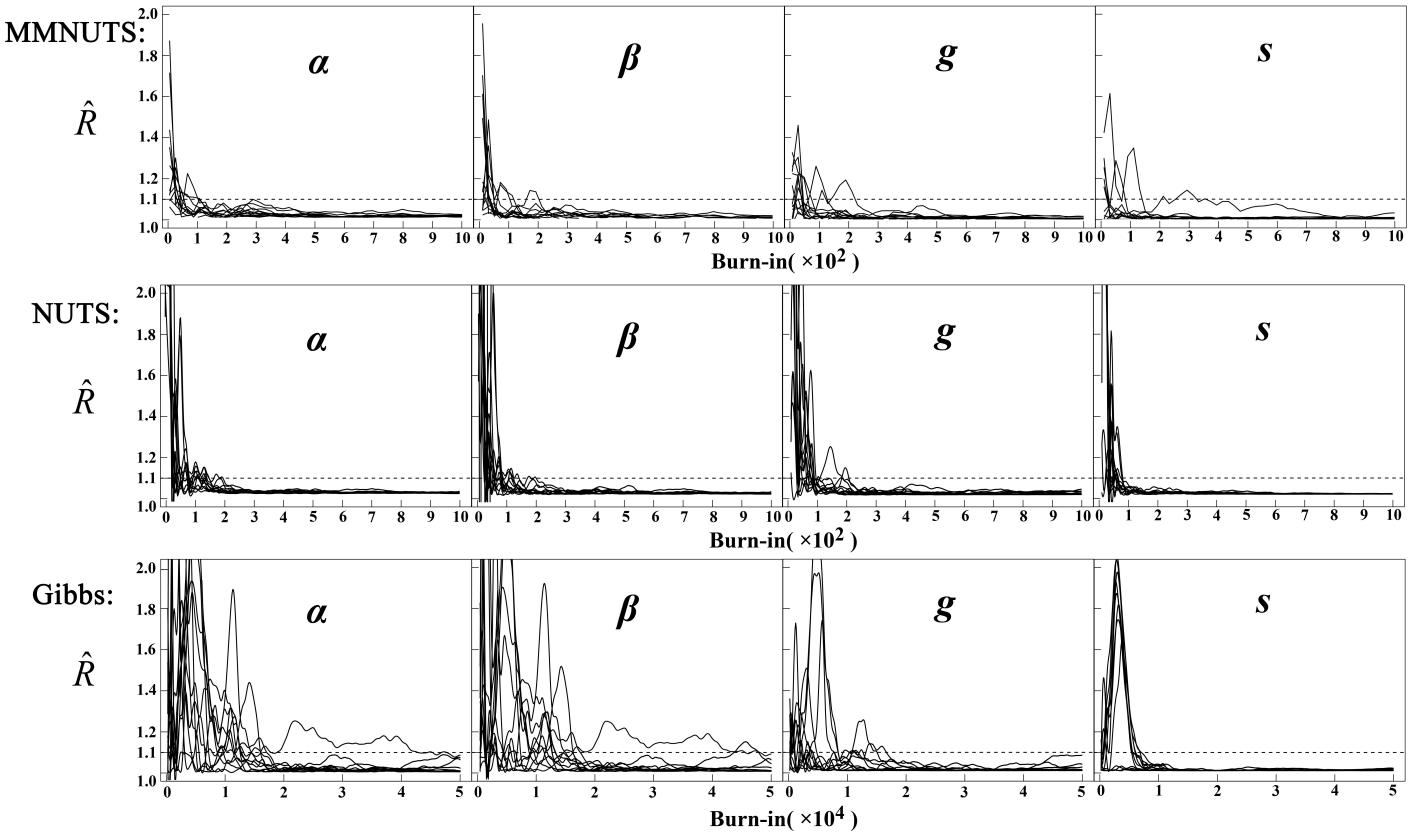
Plots of Gelman-Rubin potential scale reduction factors for the 4PNO model based on four independent chains with different starting values, Rhat, across different burn-in lengths. The Rhat plots for MMNUTS and NUTS are based upon chain lengths from 50 to 2,000 in increments of 20. The Rhat plot for the Gibbs sampler from 1,000 to 100,000 in increments of 1,000.

Figure 4 plots the Gelman-Rubin potential scale reduction factor $\hat{R}$ (Gelman and Rubin, 1992) from MMNUTS and NUTS across chain lengths from 50 to 2,000 in increments of 20 and the Gibbs-within-Gibbs sampler across chain lengths from 1,000 to 100,000 in increments of 1,000. It can be shown that MMNUTS and NUTS converge rapidly after around 1,000 iterations, but the Gibbs-within-Gibbs sampler needs approximately 50,000 iterations. These results replicate the findings of Culpepper (2015) and Carpenter et al. (2017) for the convergence rates of the No-U-Turn and Gibbs samplers, respectively.

The item estimates and corresponding standard errors produced by three samplers are displayed in Table 2, and all coefficients are comparable. However, on a 4.7GHz processor with 32GB of RAM, MMNUTS requires approximately 1.05 hours to complete a single chain (2,000 iterations), whereas fourPNO (100,000 iterations) and rstan (2,000 iterations) require 2.03 and 3.61 h, respectively. In addition to the interpretation of slipping or social desirability bias, the upper asymptote parameters for the TAU items have alternative interpretations as preference trends. For example, Items 3 to 7 have been respectively devised to detect the frequency of drinking beer, wine, liquor/spirits, pre-mixed drinks, and other drinks that contain alcohol, but a drinker may like to drink only certain beverages, resulting in a lower probability of choosing others (*s*∈[0.1820.461]). In this case, the upper asymptote parameters can be used to model the preference for alcoholic beverages.

**Table 2:** The item point estimates (Est.) with standard errors (SE) of empirical examples.

Item	*α*						*β*					
	MMNUTS		NUTS		Gibbs-within-Gibbs		MMNUTS		NUTS		Gibbs-within-Gibbs	
	Est.	SE	Est.	SE	Est.	SE	Est.	SE	Est.	SE	Est.	SE
1	2.734	0.168	2.733	0.165	2.596	0.164	3.244	0.194	3.240	0.192	3.126	0.192
2	4.041	0.112	4.011	0.116	4.349	0.251	5.887	0.138	5.840	0.142	6.382	0.349
3	3.763	0.249	3.771	0.256	3.396	0.211	2.794	0.166	2.798	0.173	2.541	0.145
4	3.878	0.369	3.891	0.409	3.145	0.201	2.289	0.208	2.297	0.235	1.843	0.111
5	6.592	0.385	6.550	0.382	5.884	0.426	5.606	0.305	5.559	0.301	5.076	0.362
6	4.983	0.364	4.955	0.364	4.370	0.317	3.635	0.262	3.606	0.264	3.206	0.227
7	5.240	0.429	5.252	0.405	4.754	0.390	3.999	0.316	4.001	0.295	3.659	0.291
8	2.354	0.109	2.362	0.105	2.302	0.128	3.225	0.134	3.232	0.131	3.179	0.160
9	4.614	0.322	4.623	0.326	4.317	0.293	3.109	0.196	3.111	0.193	2.934	0.175
10	3.586	0.220	3.601	0.234	3.546	0.183	3.485	0.181	3.497	0.190	3.470	0.147
11	3.796	0.071	3.786	0.071	4.919	0.389	5.948	0.068	5.925	0.070	7.700	0.587
12	4.031	0.311	4.045	0.338	3.764	0.265	3.955	0.276	3.963	0.300	3.745	0.236
Item	*g*						*s*					
	MMNUTS		NUTS		Gibbs-within-Gibbs		MMNUTS		NUTS		Gibbs-within-Gibbs	
	Est.	SE	Est.	SE	Est.	SE	Est.	SE	Est.	SE	Est.	SE
1	0.039	0.003	0.039	0.003	0.036	0.003	0.027	0.007	0.027	0.007	0.013	0.006
2	0.010	0.001	0.010	0.001	0.009	0.001	0.036	0.009	0.036	0.010	0.024	0.010
3	0.009	0.002	0.009	0.002	0.004	0.002	0.239	0.014	0.239	0.014	0.237	0.014
4	0.016	0.003	0.016	0.003	0.008	0.003	0.461	0.014	0.461	0.014	0.456	0.014
5	0.006	0.001	0.006	0.001	0.004	0.001	0.214	0.012	0.214	0.012	0.213	0.013
6	0.012	0.002	0.011	0.002	0.007	0.002	0.182	0.011	0.182	0.011	0.181	0.011
7	0.009	0.002	0.009	0.002	0.006	0.002	0.197	0.012	0.198	0.012	0.198	0.013
8	0.008	0.001	0.008	0.001	0.005	0.002	0.026	0.007	0.025	0.007	0.011	0.005
9	0.006	0.001	0.006	0.001	0.002	0.001	0.149	0.012	0.149	0.012	0.147	0.012
10	0.003	0.001	0.003	0.001	0.001	0.000	0.287	0.017	0.287	0.017	0.290	0.017
11	0.006	0.001	0.006	0.001	0.005	0.001	0.034	0.009	0.035	0.010	0.019	0.009
12	0.011	0.001	0.011	0.001	0.009	0.001	0.228	0.016	0.228	0.016	0.227	0.016

## 6 Discussion

The development of algorithms is an iterative process, for example, Zheng et al. (2018) developed the EMM algorithm based on Bock and Aitkin (1981)'s EM algorithm to offer a feasible MLE for the 3-parameter logistic model (3PLM) under a medium sample size; Fu et al. (2021) introduced the data augmentation scheme to the Gibbs sampler for the 4-parameter logistic model (4PLM); and Guo et al. (2023) proposed the MM-MH-RM algorithm based on Cai (2010) for the multidimensional IRT models with asymptote parameters. Therefore, this study revisited the NUT sampler from an algorithmic perspective, which may provide a foundation for future research to iteratively develop algorithms for psychometric models.

Beyond the introduction of the NUT sampler, this study further proposes a MMNUT sampler by integrating the mixture-modeling approach. It then validates the feasibility of both the original NUT sampler and the MMNUT sampler for the 4PNO model. Overall, the item recovery performance of the three samplers (MMNUTS, NUTS, and Gibbs-within-Gibbs sampler) varies across different parameters and scenarios: at times, the NUT-based approaches yield more favorable results; at other times, the Gibbs-within-Gibbs sampler performs comparably, and dedicated implementations may demonstrate stability in specific contexts. However, the NUT-based sampler has demonstrated superior time efficiency and iteration efficiency than the traditional Gibbs-based sampler, this is consistent with the previous research of Homan and Gelman (2014). As for the MMNUT sampler, although its general recovery performance is roughly comparable to those of the original NUTS, the MMNUT sample still has slight advantages in the recovery of upper asymptote parameters under weakly informative priors and larger sample sizes (≥5,000). This characteristic may be related to the design of the mixture-modeling approach. This characteristic may be attributed to the design of the mixture-modeling framework. As suggested in studies by Zheng et al. (2021) and Guo et al. (2023) on the 4PL model, the parameter estimation approach employed in the mixture model presents similar advantages in recovering the asymptotic parameters. In terms of sample size, this study also replicated the findings of Culpepper (2015) Gibbs-within-Gibbs sampler, indicating that the 4PNO model requires a sample of at least 5,000 examinees to achieve estimating stability. This finding highlights the potential value of developing optimized algorithms tailored to specific models. Another noteworthy finding is about the priors. The uniform priors for asymptote parameters may be more suitable for the educational scenario, while more informative priors may be better for the psychological scenario which has relatively larger upper or lower asymptote parameters. In addition, the priors for slope parameters also have an obvious impact on the item recovery, suggesting that researchers should choose the most appropriate priors according to specific scenarios.

Beyond the simulation studies in the fields of education and psychology, this study also examines the feasibility of applying the MMNUT algorithm and the 4PNO model to behavioral data through empirical analysis. Furthermore, analyses based on point estimates of the 4PNO model reveal that the meaning and distributional patterns of the upper and lower asymptote parameters in behavioral data are analogous to those in psychological tests, and can likewise serve as an indicator of social desirability bias. For instance, higher values of the *s* parameter suggest that individuals with higher levels of alcohol and tobacco use tend to underreport their frequency of use.

Several limitations should be noted. First, despite aiming to make the NUT sampler accessible to psychometricians via the 4PNO model, the complexity of Hamiltonian dynamics, gradient calculations, and the *BuildTree()* function still hinders easy understanding, even with step-by-step breakdowns. Second, the proposed MMNUTS shows clear advantages in running time but lacks distinct superiority in estimation accuracy. It performs comparably to the original NUT sampler and Gibbs-within-Gibbs sampler across most parameters and scenarios, with only marginal improvements in upper asymptote parameter recovery under specific conditions. Third, the exploration of the relationship between prior information and estimation accuracy is superficial. The study merely compares different prior types (more vs. less informative) in educational and psychological scenarios without delving into the mechanisms of how prior specifications (e.g., variance, distribution type) influence parameter recovery.

The following potential directions can be explored based on the current study. First, methodologically, the current study exclusively adopted the joint likelihood approach *L*(θ, *W*|*Y, Z*) for posterior distribution sampling. While this approach has demonstrated its effectiveness, future research should further expand the scope of likelihood-based methods by systematically examining the performance of conditional likelihood *L*(θ, *W, Z*|*Y*) as proposed in Culpepper (2015) under diverse scenarios. Specifically, comparisons between joint and conditional likelihood should not only focus on parameter estimation accuracy but also on computational efficiency, robustness to model misspecification, and scalability with increasing sample sizes or item numbers. Moreover, the function of the latent variable $z_{ij}^{\left(E\right)}$ is not just augmented data, the future study may try to exploit its diagnostic value in checking the ecological validity of psychological testing, especially for mental health tests.

Second, regarding stochastic-based sampling algorithms, the No-U-Turn Sampler (NUTS) employed in this study can serve as a benchmark to advance other iterative sampling methods, yet more in-depth comparisons and improvements are warranted. Future research should systematically evaluate the performance of NUTS against modified versions of classic algorithms such as Metropolis-Hastings Random Walk (MH-RM), Stochastic Approximation EM (SAEM), and Monte Carlo EM (MCEM) across multiple criteria. Additionally, exploring adaptive modifications of these algorithms (e.g., adaptive step-size adjustment for MH-RM or variance reduction techniques for SAEM) with reference to NUTS's adaptive path-finding mechanism could provide new insights into enhancing sampler performance.

Third, the extension of NUTS and its mixture-modeling revision (MMNUTS) to more complex IRT models with asymptote parameters should be further elaborated, incorporating both methodological refinements and applied validations. Beyond the five-parameter logistic (5PL) model, one-parameter logistic ability-based guessing (1PL-AG) model, two-parameter logistic extension (2PLE) model, and 4PNO with covariates model, future work should explore their multidimensional and multilevel counterparts in greater detail. For multidimensional models, it is critical to examine how MMNUTS performs in estimating correlated latent traits and handling cross-loadings, which are prevalent in multidimensional psychological assessments. For multilevel models, the adaptation of MMNUTS to account for random effects at different levels and potential heteroscedasticity across clusters is an important methodological gap to fill.

Fourth, parameter estimation under missing data should be addressed. Given that missing data are common in psychological testing due to fatigue, omitted responses, or attrition, future studies can compare MMNUTS under different missing-data strategies and develop adaptive procedures that explicitly model missing-data mechanisms. Finally, another important methodological direction involves examining the impact of violations of derivational assumptions on MMNUTS and NUTS performance. Common violations in IRT include non-normality of the latent trait distribution (e.g., skewed or bimodal distributions in clinical populations), heteroscedasticity of item residuals, and violation of local independence (e.g., item clusters or response sets). Future research should systematically simulate these assumption violations and evaluate how they affect parameter estimation accuracy, model fit indices, and sampler convergence.

## Data Availability

The original contributions presented in the study are included in the article/Supplementary material, further inquiries can be directed to the corresponding author/s.
