# Supplementary material for: The No-U-Turn sampler and its mixture-modeling revision for the 4-parameter normal ogive model
Source: Front Psychol. 2026 Mar 31;17:1746754. doi: 10.3389/fpsyg.2026.1746754 (PMC13086000; doi:10.3389/fpsyg.2026.1746754)
Supplement: Supplementary file 1 [file Supplementary_File_1.docx]

Supplementary Material

This appendix includes following four sections:

Appendix A: The MATLAB codes for the MM-No-U-Turn Sampler for the 4PNO model.

Appendix B: The mathematical derivation for MMNUTS.

Appendix C: The true item parameters in the simulation study.

Appendix D: The complete results of the simulation studies.

Appendix E: The data sources and items of tobacco and alcohol use in the empirical example.

# Appendix A: The MATLAB codes for the MM-No-U-Turn Sampler for the 4PNO model.

The function MMNUTSampler4PNO(resp,IterSize) is a user-callable MATLAB function that implements the proposed Mixture-Modeling No-U-Turn sampler to provide Bayesian estimates for the 4PNO model. This function requires the MATLAB environment and its Statistics Toolbox to operate.

The function MMNUTSampler4PNO(resp,IterSize) has two input arguments: resp and IterSize, where resp is the response matrix ($I\text{ examinees }\times\text{ }J\text{ items}$) with dichotomous (0 & 1) coding, and IterSize is a real number of MMNUTS iterations. The default priors (mean & variance) for each parameter were $\theta\sim N\left(0,1\right)$, $\log\alpha\sim N\left(0\text{，}\sqrt{0.2}\right)$, $\beta\sim N\left(0,\sqrt{2}\right)$, $\text{logit }g\sim N\left(-1.39,\sqrt{1.5}\right)$, and $\text{logit }u\sim N\left(-1.74,\sqrt{1.5}\right)$. The users can also modify these default settings in the relevant areas (see comments in the codes). Once the estimation is completed, the estimated Theta, sloping, intercept, guessing and slipping parameters with corresponding standard errors will be returned to the output arguments: Theta, alpha, beta, guess and slip, respectively.

function [Theta,alpha,beta,guess,slip,SETheta,SEalpha,SEbeta,SEguess,SEslip]=MMNUTSampler4PNO(resp,IterSize)

%resp is the response matrix

%IterSize is the number of iterations of MMNUTS

%Normal-distributed Priors for item and ability parameters

%N~[mean,variance]

%%%%Part A%%%%

thprior=[0,1]; %prior for Theta parameters

aprior=[0,0.25]; %prior for alpha parameters

bprior=[0,2]; %prior for beta parameters

gprior=[-1.39,0.25]; %prior for guessing parameters

sprior=[-1.39,0.25]; %prior for slipping parameters

%Find Reasonable Random Starting Values

[I,J]=size(resp);

[itempar0,th0]=FindReasonableStartingValues(resp,I,J);

%Initialize estimation variables

respOP=1-resp;

Colresp=mat2cell(resp,I,ones(1,J));

ColrespOP=mat2cell(respOP,I,ones(1,J));

itempar0cell=mat2cell(itempar0,4,ones(1,J));

EndWeight=[1,2];

ItemParsTotal=zeros(4,J,IterSize);

ThParsTotal=zeros(I,IterSize);

Warmupsize=floor(IterSize./2);

%Set other empirical default parameters

gamma = .05;

t0 = 10;

kappa = 0.75;

delta=0.8;

m=1;

eta0 = 1 / (m + t0);

eta1 = m^(-kappa);

%%%%Part B%%%%

%Find tuning parameters for Theta

type=0;

[EpsilonTh,EpsilonbarTh,muTh,HbarTh]=...

FindReasonableEpsilon(type,resp,respOP,th0,itempar0,I,thprior,aprior,bprior,gprior,sprior);

[th0,ALPHATh]=...

MMNUTS(type,resp,respOP,EpsilonTh,th0,itempar0,I,thprior,aprior,bprior,gprior,sprior);

[EpsilonTh,EpsilonbarTh,HbarTh]= ....

UpdateEpsilon(ALPHATh,muTh,HbarTh,EpsilonbarTh,delta,gamma,eta0,eta1,m);

ThParsTotal(:,m)=th0;

%Find tuning parameters for Item parameters

type=1;

[EpsilonItem,EpsilonbarItem,muItem,HbarItem]=cellfun(@(resp,respOP,itempar0)...

FindReasonableEpsilon(type,resp,respOP,itempar0,ThParsTotal(:,m),I,thprior,aprior,bprior,gprior,sprior),...

Colresp,ColrespOP,itempar0cell,'UniformOutput',false);

[itempar0cell,ALPHAItem]=cellfun(@(resp,respOP,itempar0,Epsilon)...

MMNUTS(type,resp,respOP,Epsilon,itempar0,ThParsTotal(:,m),I,thprior,aprior,bprior,gprior,sprior),...

Colresp,ColrespOP,itempar0cell,EpsilonItem,'UniformOutput',false);

[EpsilonItem,EpsilonbarItem,HbarItem]=cellfun(@(ALPHA,mu,Hbar,Epsilonbar) ....

UpdateEpsilon(ALPHA,mu,Hbar,Epsilonbar,delta,gamma,eta0,eta1,m),...

ALPHAItem,muItem,HbarItem,EpsilonbarItem,'UniformOutput',false);

ItemParsTotal(:,:,m)=cell2mat(itempar0cell);

%%%%Part C%%%%

%Run MMNUTS iterations

for m=2:IterSize

eta0 = 1 / (m + t0);

eta1 = m^(-kappa);

%Generating the posterior distribution for Theta

type=0;

[th0,ALPHATh]=...

MMNUTS(type,resp,respOP,EpsilonTh,th0,ItemParsTotal(:,:,m-1),I,thprior,aprior,bprior,gprior,sprior);

ThParsTotal(:,m)=th0;

%Is it Warm-up stage

if m<=Warmupsize %Yes

[EpsilonTh,EpsilonbarTh,HbarTh]= ....

UpdateEpsilon(ALPHATh,muTh,HbarTh,EpsilonbarTh,delta,gamma,eta0,eta1,m);

else %No

EpsilonTh=EpsilonbarTh;

end

%Generating the posterior distribution for Item parameters

type=1;

[itempar0cell,ALPHAItem]=cellfun(@(resp,respOP,itempar0,Epsilon)...

MMNUTS(type,resp,respOP,Epsilon,itempar0,ThParsTotal(:,m),I,thprior,aprior,bprior,gprior,sprior),...

Colresp,ColrespOP,itempar0cell,EpsilonItem,'UniformOutput',false);

%Is it Warm-up stage

if m<=Warmupsize %Yes

[EpsilonItem,EpsilonbarItem,HbarItem]=cellfun(@(ALPHA,mu,Hbar,Epsilonbar) ....

UpdateEpsilon(ALPHA,mu,Hbar,Epsilonbar,delta,gamma,eta0,eta1,m),...

ALPHAItem,muItem,HbarItem,EpsilonbarItem,'UniformOutput',false);

else %No

EpsilonItem=EpsilonbarItem;

end

ItemParsTotal(:,:,m)=cell2mat(itempar0cell);

if mod(m,100)==0 %Display the number of the current iteration

disp(m);

end

end

%Reparameterize the item parameter to IRT metric

ItemParsTotal(1,:,:)=exp(ItemParsTotal(1,:,:));

ItemParsTotal(3,:,:)=Invlogit(ItemParsTotal(3,:,:));

ItemParsTotal(4,:,:)=Invlogit(ItemParsTotal(4,:,:));

%%%%Part D%%%%

%Output the estimates and corrsponding SEs

Theta=mean(ThParsTotal(:,floor((EndWeight(1)*IterSize)/EndWeight(2)+1):IterSize),2);

alpha=mean(ItemParsTotal(1,:,floor((EndWeight(1)*IterSize)/EndWeight(2)+1):IterSize),3);

beta=mean(ItemParsTotal(2,:,floor((EndWeight(1)*IterSize)/EndWeight(2)+1):IterSize),3);

guess=mean(ItemParsTotal(3,:,floor((EndWeight(1)*IterSize)/EndWeight(2)+1):IterSize),3);

slip=mean(ItemParsTotal(4,:,floor((EndWeight(1)*IterSize)/EndWeight(2)+1):IterSize),3);

SETheta=std(ThParsTotal(:,floor((EndWeight(1)*IterSize)/EndWeight(2)+1):IterSize),1,2);

SEalpha=std(ItemParsTotal(1,:,floor((EndWeight(1)*IterSize)/EndWeight(2)+1):IterSize),1,3);

SEbeta=std(ItemParsTotal(2,:,floor((EndWeight(1)*IterSize)/EndWeight(2)+1):IterSize),1,3);

SEguess=std(ItemParsTotal(3,:,floor((EndWeight(1)*IterSize)/EndWeight(2)+1):IterSize),1,3);

SEslip=std(ItemParsTotal(4,:,floor((EndWeight(1)*IterSize)/EndWeight(2)+1):IterSize),1,3);

end

function [logp,grad]=gradFourPNO(type,resp,respOP,targetpar0,extrapar,thprior,aprior,bprior,gprior,sprior)

if type==0 %estimating for Theta parameters

A=exp(extrapar(1,:));

B=extrapar(2,:);

G=Invlogit(extrapar(3,:));

S=Invlogit(extrapar(4,:));

Phi=targetpar0*A-B;

PhiPDF=normpdf(Phi,0,1);

pstar=TwoPNO(Phi);

pgs=(1-S-G);

p4pno=G+pgs.*pstar;

q4pno=1-p4pno;

EZ=(((1-S).*pstar)./p4pno).*resp+((S.*pstar)./q4pno).*respOP;

core=(EZ-pstar)./(pstar.*(1-pstar));

Lth=core.*A.*PhiPDF;

grad=sum(Lth,2)-(targetpar0-thprior(1))/thprior(2);

logp=sum(sum(resp.*log(p4pno)+respOP.*log(q4pno),2)+log(normpdf(targetpar0,thprior(1),sqrt(thprior(2)))),1);

else %estimating for Item parameters

A=exp(targetpar0(1,:));

B=targetpar0(2,:);

G=Invlogit(targetpar0(3,:));

S=Invlogit(targetpar0(4,:));

Phi=extrapar*A-B;

PhiPDF=normpdf(Phi,0,1);

pstar=TwoPNO(Phi);

pgs=(1-S-G);

p4pno=G+pgs.*pstar;

q4pno=1-p4pno;

EZ=(((1-S).*pstar)./p4pno).*resp+((S.*pstar)./q4pno).*respOP;

core=(EZ-pstar)./(pstar.*(1-pstar));

LA=core.*extrapar.*A.*PhiPDF;

LB=-core.*PhiPDF;

LG=(resp-G).*(1-EZ);

LS=EZ.*(1-resp-S);

grad=[(sum(LA,1)-(targetpar0(1,:)-aprior(1))/aprior(2));

(sum(LB,1)-(targetpar0(2,:)-bprior(1))/bprior(2));

(sum(LG,1)-(targetpar0(3,:)-gprior(1))/gprior(2));

(sum(LS,1)-(targetpar0(4,:)-sprior(1))/sprior(2))];

logp=sum(sum(resp.*log(p4pno)+respOP.*log(q4pno),1)...

+log(normpdf(targetpar0(1,:),aprior(1),sqrt(aprior(2))))...

+log(normpdf(targetpar0(2,:),bprior(1),sqrt(bprior(2))))...

+log(normpdf(targetpar0(3,:),gprior(1),sqrt(gprior(2))))...

+log(normpdf(targetpar0(4,:),sprior(1),sqrt(sprior(2)))),2);

end

end

function [targetpar1,logp1,grad1,r1]= ...

Leapfrog(type,resp,respOP,targetpar0,extrapar,grad0,Epsilon,r0,thprior,aprior,bprior,gprior,sprior)

r1=r0+0.5.*Epsilon.*grad0;

if type==0 %estimating for Theta parameters

th1=targetpar0+Epsilon.*r1;

th1(th1>6)=6;

th1(th1<-6)=-6;

targetpar1=th1;

[logp1,grad1]=gradFourPNO(type,resp,respOP,targetpar1,extrapar,thprior,aprior,bprior,gprior,sprior);

r1=r1+0.5.*Epsilon.*grad1;

else %estimating for Item parameters

targetpar1=targetpar0+Epsilon.*r1;

logA=targetpar1(1,:);

B=targetpar1(2,:);

logitG=targetpar1(3,:);

logitS=targetpar1(4,:);

logA(logA>2)=2;

logA(logA<-5)=-5;

B(B>6)=6;

B(B<-6)=-6;

g=Invlogit(logitG);

s=Invlogit(logitS);

while (g+s)>=1

logitG=normrnd(gprior(1),sqrt(gprior(2)),1,1);

logitS=normrnd(sprior(1),sqrt(sprior(2)),1,1);

g=Invlogit(logitG);

s=Invlogit(logitS);

end

targetpar1=[logA;B;logitG;logitS];

[logp1,grad1]=gradFourPNO(type,resp,respOP,targetpar1,extrapar,thprior,aprior,bprior,gprior,sprior);

r1=r1+0.5.*Epsilon.*grad1;

end

end

function [targetparminus, rminus, gradminus, targetparplus, rplus, gradplus, ...

targetparprime, gradprime, logpprime,nprime, stopprime, alphaprime, nalphaprime]...

=BuildTree(type,targetresp,targetrespOP,targetpar,extrapar,r, grad, ...

logu, dir, depth, Epsilon, joint0, max_tree_depth,thprior,aprior,bprior,gprior,sprior)

if depth == 0

[targetparprime,logpprime,gradprime,rprime]=Leapfrog(type,targetresp,targetrespOP,targetpar,extrapar, ...

grad, dir*Epsilon, r,thprior,aprior,bprior,gprior,sprior);

joint = logpprime - 0.5 .* (rprime' * rprime);

nprime = logu < joint;

stopprime = (logu - 100) >= joint;

targetparminus = targetparprime;

targetparplus = targetparprime;

rminus = rprime;

rplus = rprime;

gradminus = gradprime;

gradplus = gradprime;

alphaprime = exp(logpprime - 0.5 * (rprime' * rprime) - joint0);

if isnan(alphaprime)

alphaprime = 0;

else

alphaprime = min(1, alphaprime);

end

nalphaprime = 1;

else

[targetparminus, rminus, gradminus, targetparplus, rplus, gradplus, ...

targetparprime, gradprime, logpprime, nprime, stopprime, alphaprime, nalphaprime] ...

=BuildTree(type,targetresp,targetrespOP,targetpar,extrapar,r, grad, ...

logu, dir, depth-1, Epsilon, joint0,max_tree_depth,thprior,aprior,bprior,gprior,sprior);

if ~stopprime

if (dir == -1)

[targetparminus, rminus, gradminus, ~, ~, ~, ...

targetparprime2, gradprime2, logpprime2, nprime2, stopprime2, alphaprime2, nalphaprime2] ...

=BuildTree(type,targetresp,targetrespOP,targetparminus,extrapar,rminus, gradminus, ...

logu, dir, depth-1, Epsilon, joint0,max_tree_depth,thprior,aprior,bprior,gprior,sprior);

else

[~, ~, ~,targetparplus, rplus, gradplus, ...

targetparprime2, gradprime2, logpprime2, nprime2, stopprime2, alphaprime2, nalphaprime2] ...

=BuildTree(type,targetresp,targetrespOP,targetparplus,extrapar,rplus, gradplus, ...

logu, dir, depth-1, Epsilon, joint0,max_tree_depth,thprior,aprior,bprior,gprior,sprior);

end

if (rand() < nprime2 / (nprime + nprime2))

targetparprime = targetparprime2;

gradprime = gradprime2;

logpprime = logpprime2;

end

nprime = nprime + nprime2;

stopprime = stopprime || stopprime2 || stopcriterion(targetparminus, targetparplus, rminus, rplus);

alphaprime = alphaprime + alphaprime2;

nalphaprime = nalphaprime + nalphaprime2;

end

end

end

function [Epsilon0,Epsilonbar,mu,Hbar]=...

FindReasonableEpsilon(type,resp,respOP,targetpar0,extrapar,I,thprior,aprior,bprior,gprior,sprior)

[logp0,grad0]=gradFourPNO(type,resp,respOP,targetpar0,extrapar,thprior,aprior,bprior,gprior,sprior);

if type==0 %estimating for Theta parameters

Epsilon0=1;

r0=normrnd(0,1,I,1);

Hbar= 0;

Epsilonbar = 1;

else %estimating for Item parameters

npar=4;

Epsilon0=ones(npar,1);

r0=normrnd(0,1,npar,1);

Hbar= zeros(npar,1);

Epsilonbar = ones(npar,1);

end

[~,logp1,~,r1]= ...

Leapfrog(type,resp,respOP,targetpar0,extrapar,grad0,Epsilon0,r0,thprior,aprior,bprior,gprior,sprior);

acceptprob = exp(logp1 - logp0 - 0.5 * (r1' * r1 - r0' * r0));

ALPHA= 2 .* (acceptprob > 0.5) - 1;

CR=(acceptprob.^ALPHA) > (2.^(-ALPHA));

ntime=1;

while(sum(CR,1)~=0 && ntime<100)

Epsilon0 = Epsilon0 .* (2.^ALPHA);

[~,logp1,~,r1]= ...

Leapfrog(type,resp,respOP,targetpar0,extrapar,grad0,Epsilon0,r0,thprior,aprior,bprior,gprior,sprior);

acceptprob = exp(logp1 - logp0 - 0.5 * (r1' * r1 - r0' * r0));

CR=(acceptprob.^ALPHA) > (2.^(-ALPHA));

ntime=ntime+1;

end

mu = log(10 * Epsilon0);

end

function [targetpar,alpha_ave]= ...

MMNUTS(type,resp,respOP,Epsilon,targetpar0,extrapar,I,thprior,aprior,bprior,gprior,sprior)

if type==0 %estimating for Theta parameters

r0=normrnd(0,1,I,1);

else %estimating for Item parameters

r0=normrnd(0,1,4,1);

end

max_tree_depth = 10;

[logp0,grad0]=gradFourPNO(type,resp,respOP,targetpar0,extrapar,thprior,aprior,bprior,gprior,sprior);

joint = logp0 - 0.5 .* (r0' * r0);

logu = joint - exprnd(1);

targetparminus = targetpar0;

targetparplus = targetpar0;

rminus = r0;

rplus = r0;

gradminus = grad0;

gradplus = grad0;

depth = 0;

targetpar = targetpar0;

n = 1;

stop = 0;

while ~stop

dir = randsample([-1,1],1);

if dir == -1

[targetparminus, rminus, gradminus, ~, ~, ~, targetparprime, ~, ~, ...

nprime, stopprime, alpha, nalpha]=BuildTree(type,resp,respOP,targetparminus,extrapar, ...

rminus, gradminus, logu, dir, depth, Epsilon, joint,max_tree_depth,thprior,aprior,bprior,gprior,sprior);

else

[~, ~, ~, targetparplus, rplus, gradplus, targetparprime, ~, ~, ...

nprime, stopprime, alpha, nalpha]=BuildTree(type,resp,respOP,targetparplus,extrapar, ...

rplus, gradplus, logu, dir, depth, Epsilon, joint,max_tree_depth,thprior,aprior,bprior,gprior,sprior);

end

if (~stopprime && (rand() < (nprime/n)))

targetpar = targetparprime;

end

n = n + nprime;

stop = stopprime | stopcriterion(targetparminus, targetparplus, rminus, rplus);

depth = depth + 1;

if depth > max_tree_depth

break;

end

end

alpha_ave = alpha / nalpha;

end

function criterion =stopcriterion(targetparminus, targetparplus, rminus, rplus)

targetparvec = targetparplus-targetparminus;

criterion = ((targetparvec' * rminus) < 0) || ((targetparvec' * rplus) < 0);

end

function P=TwoPNO(Phi)%2PNO

P=normcdf(Phi,0,1);

P(P>0.999999)=0.999999;

P(P<0.000001)=0.000001;

end

function [Epsilon,Epsilonbar,Hbar]=UpdateEpsilon(ALPHA,mu,Hbar,Epsilonbar,delta,gamma,eta0,eta1,m)

Hbar = (1 - eta0) * Hbar + eta0 * (delta - ALPHA);

Epsilon = exp(mu - sqrt(m) / gamma * Hbar);

Epsilonbar = exp((1 - eta1) * log(Epsilonbar) + eta1 * log(Epsilon));

end

function Y=Invlogit(X)%Invlogit functiion

Y=1./(1+exp(-X));

Y(Y>0.999999)=0.999999;

Y(Y<0.000001)=0.000001;

end

function Y=logit(X)

X(X==0)=0.000001;

X(X==1)=0.999999;

Y=log(X./(1-X));

end

function [itempar0,th0]=FindReasonableStartingValues(resp,I,J)

%Find the reasonable random Starting Values

CorrectRate=sum(resp,2)./J;

CorrectRate(CorrectRate>=1)=0.999;

CorrectRate(CorrectRate<=0)=0.001;

totalscore=norminv(CorrectRate,0,1);

th0=normrnd(totalscore,0.5,I,1);

th0(th0<(-2))=-2;

th0(th0>(2))=2;

respL=resp(th0<=quantile(th0,0.01),:);

g0=sum(respL,1)./size(respL,1);

logitg0=normrnd(logit(g0),0.5,1,J);

logitg0(logitg0<-2)=-2;

logitg0(logitg0>-0.2)=-0.2;

g0=Invlogit(logitg0);

respH=resp(th0>=quantile(th0,0.99),:);

s0=1-sum(respH,1)./size(respH,1);

logits0=normrnd(logit(s0),0.5,1,J);

logits0(logits0<(-2))=-2;

logits0(logits0>(-0.2))=-0.2;

s0=Invlogit(logits0);

totalscore=sum(resp,2);

pr=sum(resp,1)/I;

pr=(pr-g0)./(1-s0-g0);

pr(pr>=1)=0.999;

pr(pr<=0)=0.001;

qr=1-pr;

y=normpdf(norminv(pr,0,1),0,1);

sd=std(totalscore);

r0=zeros(1,J);

for j=1:J

x1=totalscore(resp(:,j)==1);

x2=totalscore(resp(:,j)==0);

r0(1,j)=(mean(x1,1)-mean(x2,1))./sd.*pr(j)*qr(j)./y(j);

end

r0(r0>=1)=0.9999;

r0(r0<=-1)=-0.9999;

a0=r0./sqrt(1-r0.^2);

a0(a0<0.5)=0.5;

loga0=normrnd(log(a0),0.5,1,J);

loga0(loga0<-0.5)=-0.5;

loga0(loga0>1)=1;

b0=normrnd(-norminv(pr,0,1)./sqrt(1-r0.^2),0.5,1,J);

b0(b0<-2)=-2;

b0(b0>2)=2;

itempar0=[loga0;b0;logitg0;logits0];

end

**Note: The MATLAB function MMNUTSampler4PNO.m were built and revised on the NUTS-matlab project (https://github.com/aki-nishimura/NUTS-matlab), here are the user licenses of the NUTS-matlab project:**

# License for 'NUTS.m' and 'dualAveraging.m'

Copyright (c) 2011, Matthew D. Hoffman

All rights reserved.

Redistribution and use in source and binary forms, with or without modification, are permitted provided that the following conditions are met:

Redistributions of source code must retain the above copyright notice, this list of conditions and the following disclaimer.

Redistributions in binary form must reproduce the above copyright notice, this list of conditions and the following disclaimer in the documentation and/or other materials provided with the distribution.

THIS SOFTWARE IS PROVIDED BY THE COPYRIGHT HOLDERS AND CONTRIBUTORS "AS IS" AND ANY EXPRESS OR IMPLIED WARRANTIES, INCLUDING, BUT NOT LIMITED TO, THE IMPLIED WARRANTIES OF MERCHANTABILITY AND FITNESS FOR A PARTICULAR PURPOSE ARE DISCLAIMED. IN NO EVENT SHALL THE COPYRIGHT HOLDER OR CONTRIBUTORS BE LIABLE FOR ANY DIRECT, INDIRECT, INCIDENTAL, SPECIAL, EXEMPLARY, OR CONSEQUENTIAL DAMAGES (INCLUDING, BUT NOT LIMITED TO, PROCUREMENT OF SUBSTITUTE GOODS OR SERVICES; LOSS OF USE, DATA, OR PROFITS; OR BUSINESS INTERRUPTION) HOWEVER CAUSED AND ON ANY THEORY OF LIABILITY, WHETHER IN CONTRACT, STRICT LIABILITY, OR TORT (INCLUDING NEGLIGENCE OR OTHERWISE) ARISING IN ANY WAY OUT OF THE USE OF THIS SOFTWARE, EVEN IF ADVISED OF THE POSSIBILITY OF SUCH DAMAGE.

# Licence for all the other codes in the repository.

MIT License

Copyright (c) 2016 Akihiko Nishimura

Permission is hereby granted, free of charge, to any person obtaining a copy of this software and associated documentation files (the "Software"), to deal in the Software without restriction, including without limitation the rights to use, copy, modify, merge, publish, distribute, sublicense, and/or sell copies of the Software, and to permit persons to whom the Software is furnished to do so, subject to the following conditions:

The above copyright notice and this permission notice shall be included in all copies or substantial portions of the Software.

THE SOFTWARE IS PROVIDED "AS IS", WITHOUT WARRANTY OF ANY KIND, EXPRESS OR IMPLIED, INCLUDING BUT NOT LIMITED TO THE WARRANTIES OF MERCHANTABILITY, FITNESS FOR A PARTICULAR PURPOSE AND NONINFRINGEMENT. IN NO EVENT SHALL THE AUTHORS OR COPYRIGHT HOLDERS BE LIABLE FOR ANY CLAIM, DAMAGES OR OTHER LIABILITY, WHETHER IN AN ACTION OF CONTRACT, TORT OR OTHERWISE, ARISING FROM, OUT OF OR IN CONNECTION WITH THE SOFTWARE OR THE USE OR OTHER DEALINGS IN THE SOFTWARE.

# Appendix B: The mathematical derivation for MMNUTS.

The item response function of the 4PNO model given a correct response $y_{ij}=1$ is defined by Culpepper (2015) as

$P\left(y_{ij}=1|\theta_{i},\varpi_{j}\right)\equiv P_{ij}=g_{j}+\left(1-s_{j}-g_{j}\right)P_{ij}^{*}$,

with

$P_{ij}^{*}=\Phi\left(\alpha_{j}\theta_{i}-\beta_{j}\right)$,

where $\Phi\left(\cdot\right)$ is the cumulative distribution function of the standard normal distribution; represents the response of examinee $i\left(i=1,2,...,I\right)$ on item $j\left(j=1,2,...,J\right)$; $\varpi_{j}=[\alpha_{j},\beta_{j},g_{j},s_{j}]^{T}\in\mathbf{W}$ collects the item slope $\alpha_{j}$, threshold $\beta_{j}$, lower asymptote $g_{j}$, and upper asymptote $s_{j}$ parameters for item $j$; $\theta_{i}\in\mathbf{\theta}$ is the ability parameter for subject $i$.

The mixture-modeling reformulation has been widely used in the Bayesian estimation for the IRT models with asymptotes (Culpepper, 2015; Guo et al., 2023; Guo et al., 2021; Zheng et al., 2021). Following Béguin and Glas (2001), a latent dichotomous variable has been introduced by Culpepper (2015) to provide a conditional probability of a correct response given as

,

where $z_{ij}=1$ means the examinee $i$ knows the answer of item $j$, so the unslipping probability $1-s_{j}$ will determine whether he can answer correctly; and $z_{ij}=0$ means the examinee $i$ does not know the answer of item $j$, so the guessing probability $g_{j}$ will limit the correct response. Instead of using the conditional probability in the Gibbs-within-Gibbs sampler (Culpepper, 2015), the joint probability of $\left(y_{ij},z_{ij}\right)$ was used in this study as

.

Thus, the likelihood function of $\left(y_{ij},z_{ij}\right)$ and the corresponding log-likelihood function should be

$L\left(\mathbf{\theta}\text{,}\mathbf{W}\text{|}\mathbf{Y},\mathbf{Z}\right)=\prod_{i=1}^{I} \prod_{j=1}^{J} \left[\begin{matrix} \left[\left(1-s_{j}\right)P_{ij}^{*}\right]^{y_{ij}z_{ij}}\times\left[g_{j}\left(1-P_{ij}^{*}\right)\right]^{y_{ij}\left(1-z_{ij}\right)} \\ \times\left[s_{j}P_{ij}^{*}\right]^{\left(1-y_{ij}\right)z_{ij}}\times\left[\left(1-g_{j}\right)\left(1-P_{ij}^{*}\right)\right]^{\left(1-y_{ij}\right)\left(1-z_{ij}\right)} \end{matrix}\right]$,

and

$\ln L\left(\mathbf{\theta}\text{,}\mathbf{W}\text{|}\mathbf{Y},\mathbf{Z}\right)=\sum_{i=1}^{I} \sum_{j=1}^{J} \left[\begin{matrix} z_{ij}\ln P_{ij}^{*}+\left(1-z_{ij}\right)\ln\left(1-P_{ij}^{*}\right) \\ +y_{ij}\left(1-z_{ij}\right)\ln g_{j}+\left(1-y_{ij}\right)\left(1-z_{ij}\right)\ln\left(1-g_{j}\right) \\ \left(1-y_{ij}\right)z_{ij}\ln s_{j}+y_{ij}z_{ij}\ln\left(1-s_{j}\right) \end{matrix}\right]$.

To facilitate the robustness of the sampling (Mislevy, 1986), let $\alpha_{j}^{*}=\ln\alpha_{j}$, $\gamma_{j}=\text{ln}\left(g_{j}/\left(1-g_{j}\right)\right)$, $\varsigma_{j}=\text{ln}\left(s_{j}/\left(1-s_{j}\right)\right)$, $\text{Invlogit}\left(x\right)=\left[1+\exp\left(-x\right)\right]^{-1}$, and is the probability density function of the standard normal distribution, Then, by differentiating each parameter in the above log-likelihood function, we obtain

As for the mixture-modeling reformulated 4PNO model, suppose $z_{ij}$ is known, then $L\left(\mathbf{\theta}\text{,}\mathbf{W}\text{|}\mathbf{Y},\mathbf{Z}\right)$ will be exactly equal to $L\left(\mathbf{\theta}\text{,}\mathbf{W}\text{|}\mathbf{Y}\right)$, so it is reasonable to maintain the traditional likelihood $L\left(\mathbf{\theta}\text{,}\mathbf{W}\text{|}\mathbf{Y}\right)$ for finding the optimal estimates. However, compared with other MCMC samplers that only use the ratio of log-likelihood, one significant feature of the NUT sampler is the use of gradients. In this case, the unobservable parameter $z_{ij}$ becomes a major obstacle for the NUT sampler to obtain the gradients from $L\left(\mathbf{\theta}\text{,}\mathbf{W}\text{|}\mathbf{Y},\mathbf{Z}\right)$.

Following Guo et al. (2023), this study first takes the expectation of the log-likelihood given $z_{ij}$ to obtain $E\left[\ln L\left(\mathbf{\theta}\text{,}\mathbf{W}\text{|}\mathbf{Y},\mathbf{Z}\right)\right]$, and then uses the conditional expectation $E\left(z_{ij}|y_{ij},\mathbf{\theta}_{i},\varpi_{j}\right)$ to approximate $z_{ij}$ to solve the expected log-likelihood. By Bayes rules,

$E\left(z_{ij}|y_{ij},\mathbf{\theta}_{i},\varpi_{j}\right)\equiv z_{ij}^{\left(E\right)}=\frac{\left(1-s_{j}\right)P_{ij}^{*}}{P_{ij}}\times y_{ij}+\frac{s_{j}P_{ij}^{*}}{1-P_{ij}}\times\left(1-y_{ij}\right)$.

Substitute $z_{ij}^{\left(E\right)}$ into $z_{ij}$, and further incorporate the Bayesian prior information of the normal distribution, the first derivates of MMNUTS with Bayesian priors should be:

,

,

,

$l_{\gamma_{j}}^{'}=-\frac{\gamma_{j}-\mu_{\gamma_{j}}}{\sigma_{\gamma_{j}}^{2}}+\sum_{i=1}^{I} \left(y_{ij}-\text{Invlogit}\left(\gamma_{j}\right)\right)\left(1-z_{ij}^{\left(E\right)}\right)$,

$l_{\varsigma_{j}}^{'}=-\frac{\varsigma_{j}-\mu_{\varsigma_{j}}}{\sigma_{\varsigma_{j}}^{2}}+\sum_{i=1}^{I} \left(1-y_{ij}-\text{Invlogit}\left(\varsigma_{j}\right)\right)\times z_{ij}^{\left(E\right)}$,

# Appendix C: The true item parameters in the simulation studies.

For the educational scenario:

| Item | *α* | *β* | *g* | *s* |
| --- | --- | --- | --- | --- |
| T1 | 0.747 | -0.880 | 0.202 | 0.133 |
| T2 | 0.579 | 0.145 | 0.216 | 0.252 |
| T3 | 1.218 | 0.509 | 0.086 | 0.262 |
| T4 | 0.932 | 1.328 | 0.304 | 0.190 |
| T5 | 1.258 | 0.450 | 0.175 | 0.243 |
| T6 | 0.907 | 0.370 | 0.159 | 0.210 |
| T7 | 2.248 | 1.072 | 0.265 | 0.056 |
| T8 | 2.429 | -0.410 | 0.197 | 0.148 |
| T9 | 1.786 | 0.049 | 0.101 | 0.246 |
| T10 | 1.090 | 0.266 | 0.145 | 0.145 |
| T11 | 1.075 | -1.205 | 0.112 | 0.150 |
| T12 | 1.764 | 0.180 | 0.256 | 0.129 |
| T13 | 0.835 | 0.013 | 0.152 | 0.175 |
| T14 | 1.904 | -0.923 | 0.084 | 0.170 |
| T15 | 1.705 | -1.210 | 0.138 | 0.217 |
| T16 | 2.199 | 1.637 | 0.334 | 0.091 |
| T17 | 2.347 | -1.030 | 0.083 | 0.099 |
| T18 | 0.913 | -1.918 | 0.324 | 0.211 |
| T19 | 0.752 | 0.241 | 0.306 | 0.228 |
| T20 | 2.819 | 1.479 | 0.170 | 0.127 |

For the psychological scenario:

| Item | *α* | *β* | *g* | *s* |
| --- | --- | --- | --- | --- |
| T1 | 1.91 | -0.53 | 0.04 | 0.48 |
| T2 | 1.95 | -0.31 | 0.02 | 0.52 |
| T3 | 1.5 | 0.07 | 0.02 | 0.4 |
| T4 | 1.12 | 0.07 | 0.02 | 0.37 |
| T5 | 0.89 | 0.4 | 0.04 | 0.18 |
| T6 | 1.08 | -0.54 | 0.06 | 0.17 |
| T7 | 1.16 | -0.55 | 0.07 | 0.29 |
| T8 | 1.1 | 0.01 | 0.04 | 0.27 |
| T9 | 0.78 | 0.35 | 0.05 | 0.43 |
| T10 | 1.23 | 0.23 | 0.01 | 0.1 |
| T11 | 1.34 | 0.55 | 0.02 | 0.15 |
| T12 | 1.54 | -0.74 | 0.06 | 0.41 |
| T13 | 1.16 | 0.21 | 0.02 | 0.6 |
| T14 | 0.84 | 0.6 | 0.04 | 0.25 |
| T15 | 1.13 | 0.17 | 0.03 | 0.39 |
| T16 | 0.79 | 0.94 | 0.04 | 0.27 |
| T17 | 1.27 | 0.61 | 0.01 | 0.16 |
| T18 | 0.94 | 1.29 | 0.09 | 0.06 |
| T19 | 0.84 | 1.21 | 0.02 | 0.18 |
| T20 | 1.14 | 1.73 | 0 | 0.18 |
| T21 | 1.1 | 0.28 | 0.02 | 0.07 |
| T22 | 0.72 | 0.38 | 0.24 | 0.05 |
| T23 | 0.88 | 1.37 | 0.06 | 0.09 |

# Appendix D: The complete results of the simulation studies.

For the educational scenario:

Table C.1 The bias of three samplers given 2,500 examinees for the educational scenario.

| Item | ***α*** | | | ***β*** | | | ***g*** | | | ***s*** | | |
| --- | --- | --- | --- | --- | --- | --- | --- | --- | --- | --- | --- | --- |
|  | MMNUTS | Gibbs | NUTS | MMNUTS | Gibbs | NUTS | MMNUTS | Gibbs | NUTS | MMNUTS | Gibbs | NUTS |
| T1 | 0.154 | 0.150 | 0.156 | -0.063 | -0.051 | -0.051 | 0.034 | 0.026 | 0.026 | 0.015 | 0.009 | 0.009 |
| T2 | 0.161 | 0.167 | 0.161 | 0.007 | -0.006 | -0.006 | 0.010 | -0.004 | -0.004 | 0.019 | 0.003 | 0.003 |
| T3 | 0.109 | 0.142 | 0.100 | 0.152 | 0.128 | 0.128 | 0.014 | 0.009 | 0.009 | -0.021 | -0.018 | -0.018 |
| T4 | 0.004 | 0.014 | 0.002 | -0.134 | -0.149 | -0.149 | -0.020 | -0.031 | -0.031 | 0.036 | 0.030 | 0.030 |
| T5 | -0.051 | 0.033 | -0.039 | 0.065 | 0.064 | 0.064 | -0.009 | -0.008 | -0.008 | -0.028 | -0.021 | -0.021 |
| T6 | 0.143 | 0.170 | 0.150 | 0.054 | 0.048 | 0.048 | 0.009 | 0.005 | 0.005 | 0.013 | 0.010 | 0.010 |
| T7 | 0.259 | 0.223 | 0.264 | 0.018 | 0.038 | 0.038 | -0.005 | -0.004 | -0.004 | 0.042 | 0.032 | 0.032 |
| T8 | -0.162 | -0.081 | -0.156 | 0.072 | 0.069 | 0.069 | -0.012 | -0.009 | -0.009 | -0.007 | -0.006 | -0.006 |
| T9 | 0.118 | 0.172 | 0.121 | 0.074 | 0.050 | 0.050 | 0.006 | 0.003 | 0.003 | -0.006 | -0.001 | -0.001 |
| T10 | 0.229 | 0.276 | 0.225 | 0.039 | 0.042 | 0.042 | 0.018 | 0.017 | 0.017 | 0.035 | 0.034 | 0.034 |
| T11 | 0.189 | 0.230 | 0.189 | 0.049 | 0.037 | 0.037 | 0.092 | 0.094 | 0.094 | 0.002 | 0.002 | 0.002 |
| T12 | 0.030 | 0.124 | 0.034 | -0.003 | 0.020 | 0.020 | -0.015 | -0.010 | -0.010 | 0.005 | 0.003 | 0.003 |
| T13 | 0.221 | 0.261 | 0.215 | 0.063 | 0.060 | 0.060 | 0.037 | 0.035 | 0.035 | 0.027 | 0.025 | 0.025 |
| T14 | 0.336 | 0.340 | 0.338 | 0.032 | 0.008 | 0.008 | 0.038 | 0.031 | 0.031 | 0.000 | 0.001 | 0.001 |
| T15 | 0.147 | 0.244 | 0.154 | 0.056 | 0.005 | 0.005 | 0.024 | 0.025 | 0.025 | -0.005 | -0.003 | -0.003 |
| T16 | 0.035 | 0.108 | 0.061 | -0.110 | -0.040 | -0.040 | -0.011 | -0.009 | -0.009 | 0.043 | 0.037 | 0.037 |
| T17 | 0.418 | 0.336 | 0.410 | -0.001 | 0.016 | 0.016 | 0.031 | 0.024 | 0.024 | 0.005 | 0.002 | 0.002 |
| T18 | -0.003 | -0.050 | -0.007 | -0.098 | -0.024 | -0.024 | -0.099 | -0.109 | -0.109 | -0.009 | -0.016 | -0.016 |
| T19 | 0.012 | -0.001 | 0.019 | -0.090 | -0.093 | -0.093 | -0.038 | -0.053 | -0.053 | 0.001 | -0.013 | -0.013 |
| T20 | -0.299 | -0.270 | -0.298 | -0.104 | -0.081 | -0.081 | -0.007 | -0.006 | -0.006 | 0.005 | 0.000 | 0.000 |

Table C.2 The RMSEs of three samplers given 2,500 examinees for the educational scenario.

| Item | ***α*** | | | ***β*** | | | ***g*** | | | ***s*** | | |
| --- | --- | --- | --- | --- | --- | --- | --- | --- | --- | --- | --- | --- |
|  | MMNUTS | Gibbs | NUTS | MMNUTS | Gibbs | NUTS | MMNUTS | Gibbs | NUTS | MMNUTS | Gibbs | NUTS |
| T1 | 0.198 | 0.222 | 0.200 | 0.193 | 0.212 | 0.192 | 0.044 | 0.038 | 0.041 | 0.025 | 0.026 | 0.025 |
| T2 | 0.203 | 0.327 | 0.207 | 0.245 | 0.267 | 0.252 | 0.032 | 0.035 | 0.033 | 0.042 | 0.044 | 0.043 |
| T3 | 0.229 | 0.314 | 0.213 | 0.207 | 0.197 | 0.206 | 0.019 | 0.018 | 0.019 | 0.047 | 0.053 | 0.048 |
| T4 | 0.189 | 0.292 | 0.195 | 0.332 | 0.413 | 0.333 | 0.030 | 0.042 | 0.030 | 0.041 | 0.035 | 0.039 |
| T5 | 0.185 | 0.257 | 0.190 | 0.148 | 0.160 | 0.150 | 0.021 | 0.025 | 0.022 | 0.042 | 0.044 | 0.040 |
| T6 | 0.224 | 0.308 | 0.236 | 0.155 | 0.162 | 0.155 | 0.022 | 0.026 | 0.023 | 0.039 | 0.042 | 0.039 |
| T7 | 0.453 | 0.390 | 0.455 | 0.234 | 0.218 | 0.235 | 0.017 | 0.016 | 0.017 | 0.044 | 0.035 | 0.044 |
| T8 | 0.461 | 0.422 | 0.456 | 0.130 | 0.135 | 0.128 | 0.028 | 0.029 | 0.028 | 0.017 | 0.018 | 0.017 |
| T9 | 0.472 | 0.473 | 0.460 | 0.161 | 0.157 | 0.161 | 0.017 | 0.019 | 0.017 | 0.031 | 0.032 | 0.032 |
| T10 | 0.309 | 0.381 | 0.302 | 0.138 | 0.149 | 0.136 | 0.028 | 0.031 | 0.028 | 0.043 | 0.046 | 0.043 |
| T11 | 0.253 | 0.306 | 0.253 | 0.190 | 0.202 | 0.187 | 0.095 | 0.098 | 0.095 | 0.014 | 0.015 | 0.014 |
| T12 | 0.380 | 0.453 | 0.381 | 0.131 | 0.134 | 0.129 | 0.028 | 0.026 | 0.028 | 0.020 | 0.024 | 0.020 |
| T13 | 0.260 | 0.328 | 0.251 | 0.155 | 0.169 | 0.154 | 0.043 | 0.044 | 0.043 | 0.041 | 0.044 | 0.039 |
| T14 | 0.452 | 0.446 | 0.445 | 0.190 | 0.187 | 0.185 | 0.041 | 0.035 | 0.040 | 0.018 | 0.018 | 0.018 |
| T15 | 0.282 | 0.361 | 0.287 | 0.214 | 0.217 | 0.210 | 0.036 | 0.040 | 0.035 | 0.018 | 0.017 | 0.018 |
| T16 | 0.324 | 0.356 | 0.324 | 0.296 | 0.296 | 0.294 | 0.018 | 0.017 | 0.018 | 0.047 | 0.043 | 0.048 |
| T17 | 0.547 | 0.453 | 0.536 | 0.162 | 0.150 | 0.158 | 0.034 | 0.029 | 0.034 | 0.011 | 0.011 | 0.011 |
| T18 | 0.180 | 0.273 | 0.177 | 0.329 | 0.383 | 0.321 | 0.100 | 0.110 | 0.102 | 0.017 | 0.023 | 0.017 |
| T19 | 0.128 | 0.216 | 0.134 | 0.250 | 0.250 | 0.251 | 0.053 | 0.069 | 0.053 | 0.030 | 0.034 | 0.031 |
| T20 | 0.456 | 0.405 | 0.442 | 0.237 | 0.206 | 0.230 | 0.015 | 0.015 | 0.015 | 0.018 | 0.021 | 0.017 |

Table C.3 The PSDs of three samplers given 2,500 examinees for the educational scenario.

| Item | ***α*** | | | ***β*** | | | ***g*** | | | ***s*** | | |
| --- | --- | --- | --- | --- | --- | --- | --- | --- | --- | --- | --- | --- |
|  | MMNUTS | Gibbs | NUTS | MMNUTS | Gibbs | NUTS | MMNUTS | Gibbs | NUTS | MMNUTS | Gibbs | NUTS |
| T1 | 0.223 | 0.257 | 0.226 | 0.225 | 0.236 | 0.236 | 0.085 | 0.093 | 0.093 | 0.024 | 0.028 | 0.028 |
| T2 | 0.295 | 0.359 | 0.294 | 0.317 | 0.311 | 0.311 | 0.058 | 0.066 | 0.066 | 0.080 | 0.088 | 0.088 |
| T3 | 0.304 | 0.347 | 0.296 | 0.150 | 0.157 | 0.157 | 0.018 | 0.021 | 0.021 | 0.064 | 0.069 | 0.069 |
| T4 | 0.330 | 0.394 | 0.325 | 0.404 | 0.440 | 0.440 | 0.034 | 0.044 | 0.044 | 0.081 | 0.090 | 0.090 |
| T5 | 0.316 | 0.381 | 0.326 | 0.173 | 0.184 | 0.184 | 0.029 | 0.031 | 0.031 | 0.059 | 0.065 | 0.065 |
| T6 | 0.280 | 0.318 | 0.284 | 0.186 | 0.194 | 0.194 | 0.033 | 0.038 | 0.038 | 0.063 | 0.070 | 0.070 |
| T7 | 0.559 | 0.505 | 0.559 | 0.271 | 0.249 | 0.249 | 0.018 | 0.018 | 0.018 | 0.024 | 0.027 | 0.027 |
| T8 | 0.539 | 0.517 | 0.553 | 0.145 | 0.149 | 0.149 | 0.030 | 0.031 | 0.031 | 0.019 | 0.019 | 0.019 |
| T9 | 0.441 | 0.447 | 0.445 | 0.141 | 0.145 | 0.145 | 0.020 | 0.023 | 0.023 | 0.035 | 0.036 | 0.036 |
| T10 | 0.296 | 0.351 | 0.293 | 0.151 | 0.160 | 0.160 | 0.030 | 0.033 | 0.033 | 0.044 | 0.049 | 0.049 |
| T11 | 0.286 | 0.322 | 0.285 | 0.225 | 0.236 | 0.236 | 0.068 | 0.077 | 0.077 | 0.019 | 0.020 | 0.020 |
| T12 | 0.436 | 0.467 | 0.446 | 0.158 | 0.163 | 0.163 | 0.032 | 0.032 | 0.032 | 0.027 | 0.030 | 0.030 |
| T13 | 0.267 | 0.335 | 0.271 | 0.179 | 0.187 | 0.187 | 0.043 | 0.050 | 0.050 | 0.048 | 0.055 | 0.055 |
| T14 | 0.456 | 0.450 | 0.463 | 0.196 | 0.198 | 0.198 | 0.030 | 0.034 | 0.034 | 0.017 | 0.017 | 0.017 |
| T15 | 0.444 | 0.465 | 0.447 | 0.259 | 0.261 | 0.261 | 0.045 | 0.051 | 0.051 | 0.018 | 0.018 | 0.018 |
| T16 | 0.548 | 0.521 | 0.574 | 0.364 | 0.347 | 0.347 | 0.017 | 0.016 | 0.016 | 0.036 | 0.040 | 0.040 |
| T17 | 0.552 | 0.501 | 0.552 | 0.205 | 0.191 | 0.191 | 0.026 | 0.029 | 0.029 | 0.013 | 0.013 | 0.013 |
| T18 | 0.298 | 0.347 | 0.298 | 0.471 | 0.538 | 0.538 | 0.088 | 0.093 | 0.093 | 0.017 | 0.025 | 0.025 |
| T19 | 0.294 | 0.345 | 0.301 | 0.310 | 0.311 | 0.311 | 0.067 | 0.077 | 0.077 | 0.066 | 0.074 | 0.074 |
| T20 | 0.544 | 0.518 | 0.543 | 0.275 | 0.259 | 0.259 | 0.014 | 0.014 | 0.014 | 0.033 | 0.037 | 0.037 |

Table C.4 The bias of three samplers given 5,000 examinees for the educational scenario.

| Item | ***α*** | | | ***β*** | | | ***g*** | | | ***s*** | | |
| --- | --- | --- | --- | --- | --- | --- | --- | --- | --- | --- | --- | --- |
|  | MMNUTS | Gibbs | NUTS | MMNUTS | Gibbs | NUTS | MMNUTS | Gibbs | NUTS | MMNUTS | Gibbs | NUTS |
| T1 | 0.090 | 0.068 | 0.090 | -0.017 | -0.001 | -0.001 | 0.027 | 0.017 | 0.017 | 0.008 | 0.002 | 0.002 |
| T2 | 0.109 | 0.064 | 0.103 | 0.028 | 0.020 | 0.020 | 0.009 | -0.009 | -0.009 | 0.004 | -0.016 | -0.016 |
| T3 | 0.073 | 0.100 | 0.068 | 0.082 | 0.061 | 0.061 | 0.008 | 0.005 | 0.005 | -0.014 | -0.011 | -0.011 |
| T4 | 0.101 | 0.091 | 0.096 | 0.038 | 0.033 | 0.033 | -0.006 | -0.010 | -0.010 | 0.027 | 0.018 | 0.018 |
| T5 | -0.036 | 0.030 | -0.035 | 0.031 | 0.033 | 0.033 | -0.007 | -0.005 | -0.005 | -0.022 | -0.017 | -0.017 |
| T6 | 0.073 | 0.099 | 0.078 | 0.021 | 0.013 | 0.013 | 0.005 | 0.003 | 0.003 | 0.005 | 0.007 | 0.007 |
| T7 | 0.276 | 0.182 | 0.281 | 0.041 | 0.035 | 0.035 | -0.004 | -0.004 | -0.004 | 0.027 | 0.017 | 0.017 |
| T8 | -0.088 | -0.029 | -0.096 | 0.025 | 0.020 | 0.020 | -0.009 | -0.007 | -0.007 | -0.003 | -0.003 | -0.003 |
| T9 | 0.028 | 0.093 | 0.021 | 0.058 | 0.044 | 0.044 | 0.003 | 0.001 | 0.001 | -0.010 | -0.007 | -0.007 |
| T10 | 0.111 | 0.121 | 0.111 | 0.019 | 0.017 | 0.017 | 0.007 | 0.005 | 0.005 | 0.017 | 0.015 | 0.015 |
| T11 | 0.166 | 0.188 | 0.166 | 0.037 | 0.025 | 0.025 | 0.081 | 0.081 | 0.081 | 0.001 | 0.002 | 0.002 |
| T12 | 0.018 | 0.084 | 0.015 | -0.008 | 0.009 | 0.009 | -0.008 | -0.004 | -0.004 | 0.002 | 0.002 | 0.002 |
| T13 | 0.124 | 0.122 | 0.127 | -0.013 | -0.016 | -0.016 | 0.016 | 0.010 | 0.010 | 0.023 | 0.018 | 0.018 |
| T14 | 0.268 | 0.234 | 0.263 | -0.043 | -0.051 | -0.051 | 0.025 | 0.016 | 0.016 | 0.004 | 0.004 | 0.004 |
| T15 | 0.082 | 0.130 | 0.069 | 0.057 | 0.028 | 0.028 | 0.015 | 0.014 | 0.014 | -0.004 | -0.003 | -0.003 |
| T16 | 0.168 | 0.132 | 0.162 | 0.007 | 0.013 | 0.013 | -0.003 | -0.003 | -0.003 | 0.033 | 0.024 | 0.024 |
| T17 | 0.250 | 0.166 | 0.252 | -0.012 | 0.003 | 0.003 | 0.020 | 0.012 | 0.012 | 0.003 | 0.002 | 0.002 |
| T18 | -0.034 | -0.092 | -0.036 | -0.078 | 0.023 | 0.023 | -0.099 | -0.104 | -0.104 | -0.004 | -0.011 | -0.011 |
| T19 | -0.017 | -0.035 | -0.016 | -0.068 | -0.076 | -0.076 | -0.039 | -0.053 | -0.053 | -0.017 | -0.030 | -0.030 |
| T20 | -0.065 | -0.158 | -0.078 | 0.021 | -0.007 | -0.007 | -0.002 | -0.002 | -0.002 | -0.004 | -0.012 | -0.012 |

Table C.5 The RMSEs of three samplers given 5,000 examinees for the educational scenario.

| Item | ***α*** | | | ***β*** | | | ***g*** | | | ***s*** | | |
| --- | --- | --- | --- | --- | --- | --- | --- | --- | --- | --- | --- | --- |
|  | MMNUTS | Gibbs | NUTS | MMNUTS | Gibbs | NUTS | MMNUTS | Gibbs | NUTS | MMNUTS | Gibbs | NUTS |
| T1 | 0.128 | 0.137 | 0.128 | 0.162 | 0.172 | 0.161 | 0.041 | 0.034 | 0.039 | 0.019 | 0.021 | 0.019 |
| T2 | 0.132 | 0.140 | 0.122 | 0.208 | 0.202 | 0.208 | 0.030 | 0.034 | 0.029 | 0.040 | 0.042 | 0.037 |
| T3 | 0.181 | 0.248 | 0.175 | 0.131 | 0.123 | 0.130 | 0.016 | 0.017 | 0.015 | 0.043 | 0.049 | 0.043 |
| T4 | 0.226 | 0.267 | 0.219 | 0.314 | 0.364 | 0.306 | 0.019 | 0.025 | 0.019 | 0.032 | 0.023 | 0.029 |
| T5 | 0.212 | 0.286 | 0.217 | 0.111 | 0.118 | 0.116 | 0.021 | 0.023 | 0.021 | 0.042 | 0.047 | 0.043 |
| T6 | 0.142 | 0.185 | 0.142 | 0.103 | 0.106 | 0.104 | 0.018 | 0.020 | 0.017 | 0.029 | 0.035 | 0.030 |
| T7 | 0.420 | 0.359 | 0.431 | 0.182 | 0.174 | 0.184 | 0.015 | 0.015 | 0.015 | 0.029 | 0.021 | 0.029 |
| T8 | 0.360 | 0.348 | 0.363 | 0.101 | 0.104 | 0.103 | 0.022 | 0.022 | 0.022 | 0.013 | 0.014 | 0.014 |
| T9 | 0.268 | 0.351 | 0.258 | 0.109 | 0.107 | 0.109 | 0.016 | 0.018 | 0.015 | 0.029 | 0.030 | 0.029 |
| T10 | 0.188 | 0.219 | 0.190 | 0.090 | 0.096 | 0.091 | 0.021 | 0.023 | 0.021 | 0.026 | 0.029 | 0.026 |
| T11 | 0.209 | 0.238 | 0.205 | 0.148 | 0.156 | 0.149 | 0.085 | 0.087 | 0.086 | 0.013 | 0.013 | 0.012 |
| T12 | 0.298 | 0.321 | 0.298 | 0.086 | 0.091 | 0.087 | 0.024 | 0.023 | 0.024 | 0.019 | 0.021 | 0.018 |
| T13 | 0.172 | 0.196 | 0.170 | 0.124 | 0.131 | 0.125 | 0.027 | 0.030 | 0.028 | 0.039 | 0.040 | 0.038 |
| T14 | 0.391 | 0.353 | 0.379 | 0.151 | 0.148 | 0.147 | 0.028 | 0.024 | 0.028 | 0.014 | 0.014 | 0.014 |
| T15 | 0.258 | 0.302 | 0.244 | 0.171 | 0.170 | 0.171 | 0.026 | 0.030 | 0.025 | 0.012 | 0.011 | 0.012 |
| T16 | 0.379 | 0.329 | 0.374 | 0.261 | 0.232 | 0.256 | 0.011 | 0.010 | 0.011 | 0.038 | 0.032 | 0.038 |
| T17 | 0.413 | 0.345 | 0.428 | 0.141 | 0.127 | 0.141 | 0.025 | 0.022 | 0.025 | 0.010 | 0.010 | 0.010 |
| T18 | 0.176 | 0.254 | 0.176 | 0.317 | 0.385 | 0.310 | 0.099 | 0.104 | 0.101 | 0.013 | 0.020 | 0.012 |
| T19 | 0.141 | 0.214 | 0.138 | 0.199 | 0.197 | 0.193 | 0.054 | 0.069 | 0.053 | 0.032 | 0.043 | 0.030 |
| T20 | 0.396 | 0.388 | 0.412 | 0.188 | 0.172 | 0.196 | 0.010 | 0.011 | 0.011 | 0.020 | 0.025 | 0.020 |

Table C.6 The PSDs of three samplers given 5,000 examinees for the educational scenario.

| Item | ***α*** | | | ***β*** | | | ***g*** | | | ***s*** | | |
| --- | --- | --- | --- | --- | --- | --- | --- | --- | --- | --- | --- | --- |
|  | MMNUTS | Gibbs | NUTS | MMNUTS | Gibbs | NUTS | MMNUTS | Gibbs | NUTS | MMNUTS | Gibbs | NUTS |
| T1 | 0.164 | 0.180 | 0.168 | 0.168 | 0.170 | 0.170 | 0.079 | 0.086 | 0.086 | 0.021 | 0.023 | 0.023 |
| T2 | 0.227 | 0.238 | 0.228 | 0.239 | 0.237 | 0.237 | 0.053 | 0.061 | 0.061 | 0.073 | 0.081 | 0.081 |
| T3 | 0.239 | 0.277 | 0.238 | 0.109 | 0.113 | 0.113 | 0.015 | 0.017 | 0.017 | 0.055 | 0.061 | 0.061 |
| T4 | 0.295 | 0.307 | 0.293 | 0.329 | 0.331 | 0.331 | 0.021 | 0.024 | 0.024 | 0.074 | 0.082 | 0.082 |
| T5 | 0.267 | 0.307 | 0.268 | 0.126 | 0.132 | 0.132 | 0.023 | 0.024 | 0.024 | 0.052 | 0.056 | 0.056 |
| T6 | 0.208 | 0.247 | 0.213 | 0.138 | 0.142 | 0.142 | 0.029 | 0.033 | 0.033 | 0.057 | 0.064 | 0.064 |
| T7 | 0.440 | 0.383 | 0.442 | 0.202 | 0.179 | 0.179 | 0.013 | 0.013 | 0.013 | 0.019 | 0.021 | 0.021 |
| T8 | 0.446 | 0.418 | 0.449 | 0.107 | 0.109 | 0.109 | 0.023 | 0.023 | 0.023 | 0.014 | 0.014 | 0.014 |
| T9 | 0.308 | 0.349 | 0.313 | 0.095 | 0.100 | 0.100 | 0.016 | 0.018 | 0.018 | 0.026 | 0.026 | 0.026 |
| T10 | 0.196 | 0.226 | 0.197 | 0.105 | 0.107 | 0.107 | 0.025 | 0.028 | 0.028 | 0.037 | 0.043 | 0.043 |
| T11 | 0.215 | 0.243 | 0.218 | 0.158 | 0.165 | 0.165 | 0.062 | 0.069 | 0.069 | 0.014 | 0.014 | 0.014 |
| T12 | 0.338 | 0.355 | 0.344 | 0.109 | 0.113 | 0.113 | 0.023 | 0.023 | 0.023 | 0.022 | 0.023 | 0.023 |
| T13 | 0.186 | 0.209 | 0.189 | 0.132 | 0.134 | 0.134 | 0.038 | 0.044 | 0.044 | 0.042 | 0.047 | 0.047 |
| T14 | 0.338 | 0.335 | 0.338 | 0.149 | 0.141 | 0.141 | 0.025 | 0.028 | 0.028 | 0.012 | 0.012 | 0.012 |
| T15 | 0.326 | 0.338 | 0.321 | 0.178 | 0.179 | 0.179 | 0.039 | 0.044 | 0.044 | 0.013 | 0.013 | 0.013 |
| T16 | 0.487 | 0.468 | 0.496 | 0.307 | 0.283 | 0.283 | 0.012 | 0.012 | 0.012 | 0.030 | 0.035 | 0.035 |
| T17 | 0.391 | 0.358 | 0.397 | 0.147 | 0.136 | 0.136 | 0.022 | 0.024 | 0.024 | 0.009 | 0.009 | 0.009 |
| T18 | 0.263 | 0.285 | 0.260 | 0.416 | 0.435 | 0.435 | 0.087 | 0.096 | 0.096 | 0.014 | 0.020 | 0.020 |
| T19 | 0.235 | 0.255 | 0.235 | 0.228 | 0.224 | 0.224 | 0.059 | 0.066 | 0.066 | 0.059 | 0.067 | 0.067 |
| T20 | 0.498 | 0.429 | 0.496 | 0.237 | 0.206 | 0.206 | 0.010 | 0.010 | 0.010 | 0.026 | 0.028 | 0.028 |

For the psychological scenario:

Table C.7 The bias of three samplers given 2,500 examinees for the psychological scenario.

| Item | ***α*** | | | ***β*** | | | ***g*** | | | ***s*** | | |
| --- | --- | --- | --- | --- | --- | --- | --- | --- | --- | --- | --- | --- |
|  | MMNUTS | Gibbs | NUTS | MMNUTS | Gibbs | NUTS | MMNUTS | Gibbs | NUTS | MMNUTS | Gibbs | NUTS |
| T1 | 0.145 | 0.109 | 0.053 | 0.093 | 0.041 | 0.041 | 0.014 | 0.003 | 0.003 | -0.011 | -0.009 | -0.009 |
| T2 | 0.246 | 0.147 | -0.141 | 0.112 | 0.046 | 0.046 | 0.016 | 0.004 | 0.004 | -0.010 | -0.008 | -0.008 |
| T3 | 0.265 | 0.194 | 0.263 | 0.099 | 0.039 | 0.039 | 0.019 | 0.007 | 0.007 | -0.008 | -0.009 | -0.009 |
| T4 | 0.266 | 0.207 | 0.266 | 0.106 | 0.053 | 0.053 | 0.033 | 0.017 | 0.017 | -0.008 | -0.012 | -0.012 |
| T5 | 0.196 | 0.138 | 0.196 | 0.084 | 0.053 | 0.053 | 0.030 | 0.014 | 0.014 | 0.016 | -0.005 | -0.005 |
| T6 | 0.194 | 0.169 | 0.195 | 0.044 | 0.031 | 0.031 | 0.050 | 0.033 | 0.033 | 0.001 | -0.003 | -0.003 |
| T7 | 0.211 | 0.243 | 0.209 | 0.046 | 0.015 | 0.015 | 0.036 | 0.024 | 0.024 | -0.007 | -0.007 | -0.007 |
| T8 | 0.183 | 0.152 | 0.182 | 0.088 | 0.053 | 0.053 | 0.028 | 0.013 | 0.013 | -0.011 | -0.017 | -0.017 |
| T9 | 0.094 | 0.027 | 0.019 | 0.279 | 0.240 | 0.240 | 0.023 | 0.003 | 0.003 | -0.099 | -0.137 | -0.137 |
| T10 | 0.258 | 0.159 | 0.258 | 0.086 | 0.055 | 0.055 | 0.034 | 0.018 | 0.018 | 0.018 | 0.003 | 0.003 |
| T11 | 0.209 | 0.116 | 0.214 | 0.081 | 0.039 | 0.039 | 0.017 | 0.005 | 0.005 | 0.006 | -0.009 | -0.009 |
| T12 | 0.170 | 0.133 | 0.156 | 0.068 | 0.032 | 0.032 | 0.022 | 0.007 | 0.007 | -0.011 | -0.011 | -0.011 |
| T13 | 0.164 | 0.091 | -0.328 | 0.267 | 0.189 | 0.189 | 0.018 | 0.004 | 0.004 | -0.085 | -0.101 | -0.101 |
| T14 | 0.170 | 0.107 | 0.171 | 0.090 | 0.045 | 0.045 | 0.019 | 0.004 | 0.004 | 0.000 | -0.024 | -0.024 |
| T15 | 0.183 | 0.125 | 0.171 | 0.135 | 0.082 | 0.082 | 0.022 | 0.006 | 0.006 | -0.028 | -0.035 | -0.035 |
| T16 | 0.135 | 0.071 | 0.111 | 0.102 | 0.037 | 0.037 | 0.013 | 0.000 | 0.000 | -0.013 | -0.039 | -0.039 |
| T17 | 0.252 | 0.151 | 0.252 | 0.101 | 0.050 | 0.050 | 0.020 | 0.008 | 0.008 | 0.014 | -0.001 | -0.001 |
| T18 | 0.227 | 0.242 | 0.229 | -0.005 | 0.004 | 0.004 | 0.004 | 0.002 | 0.002 | 0.116 | 0.103 | 0.103 |
| T19 | 0.220 | 0.146 | 0.203 | 0.101 | 0.034 | 0.034 | 0.014 | 0.005 | 0.005 | 0.056 | 0.027 | 0.027 |
| T20 | 0.275 | 0.193 | 0.272 | 0.201 | 0.088 | 0.088 | 0.010 | 0.004 | 0.004 | 0.045 | 0.032 | 0.032 |
| T21 | 0.249 | 0.155 | 0.249 | 0.063 | 0.040 | 0.040 | 0.036 | 0.019 | 0.019 | 0.034 | 0.014 | 0.014 |
| T22 | 0.194 | 0.170 | 0.197 | -0.102 | -0.078 | -0.078 | -0.008 | -0.020 | -0.020 | 0.073 | 0.048 | 0.048 |
| T23 | 0.207 | 0.205 | 0.210 | 0.020 | 0.002 | 0.002 | 0.006 | 0.002 | 0.002 | 0.108 | 0.095 | 0.095 |

Table C.8 The RMSEs of three samplers given 2,500 examinees for the psychological scenario.

| Item | ***α*** | | | ***β*** | | | ***g*** | | | ***s*** | | |
| --- | --- | --- | --- | --- | --- | --- | --- | --- | --- | --- | --- | --- |
|  | MMNUTS | Gibbs | NUTS | MMNUTS | Gibbs | NUTS | MMNUTS | Gibbs | NUTS | MMNUTS | Gibbs | NUTS |
| T1 | 0.389 | 0.380 | 0.313 | 0.224 | 0.203 | 0.219 | 0.018 | 0.017 | 0.018 | 0.026 | 0.026 | 0.025 |
| T2 | 0.451 | 0.388 | 0.314 | 0.209 | 0.179 | 0.317 | 0.018 | 0.010 | 0.016 | 0.030 | 0.029 | 0.045 |
| T3 | 0.386 | 0.366 | 0.378 | 0.163 | 0.140 | 0.163 | 0.021 | 0.013 | 0.021 | 0.035 | 0.037 | 0.035 |
| T4 | 0.348 | 0.322 | 0.346 | 0.191 | 0.173 | 0.190 | 0.034 | 0.021 | 0.034 | 0.049 | 0.056 | 0.049 |
| T5 | 0.236 | 0.227 | 0.231 | 0.141 | 0.130 | 0.141 | 0.034 | 0.025 | 0.034 | 0.050 | 0.057 | 0.048 |
| T6 | 0.250 | 0.263 | 0.249 | 0.131 | 0.131 | 0.133 | 0.054 | 0.044 | 0.054 | 0.026 | 0.030 | 0.027 |
| T7 | 0.331 | 0.401 | 0.331 | 0.199 | 0.198 | 0.202 | 0.042 | 0.041 | 0.042 | 0.033 | 0.036 | 0.034 |
| T8 | 0.258 | 0.290 | 0.265 | 0.156 | 0.147 | 0.159 | 0.031 | 0.024 | 0.032 | 0.045 | 0.053 | 0.045 |
| T9 | 0.186 | 0.252 | 0.116 | 0.323 | 0.292 | 0.352 | 0.027 | 0.020 | 0.026 | 0.119 | 0.158 | 0.137 |
| T10 | 0.295 | 0.226 | 0.294 | 0.110 | 0.092 | 0.110 | 0.035 | 0.022 | 0.035 | 0.031 | 0.032 | 0.031 |
| T11 | 0.250 | 0.197 | 0.257 | 0.120 | 0.102 | 0.122 | 0.019 | 0.011 | 0.019 | 0.036 | 0.045 | 0.037 |
| T12 | 0.333 | 0.334 | 0.315 | 0.211 | 0.201 | 0.213 | 0.026 | 0.021 | 0.025 | 0.028 | 0.029 | 0.028 |
| T13 | 0.432 | 0.469 | 0.341 | 0.386 | 0.352 | 0.571 | 0.019 | 0.010 | 0.015 | 0.137 | 0.161 | 0.229 |
| T14 | 0.209 | 0.195 | 0.210 | 0.156 | 0.138 | 0.152 | 0.022 | 0.016 | 0.022 | 0.059 | 0.074 | 0.057 |
| T15 | 0.299 | 0.307 | 0.285 | 0.195 | 0.170 | 0.196 | 0.024 | 0.016 | 0.024 | 0.061 | 0.073 | 0.062 |
| T16 | 0.180 | 0.172 | 0.145 | 0.169 | 0.149 | 0.164 | 0.017 | 0.014 | 0.016 | 0.058 | 0.079 | 0.051 |
| T17 | 0.303 | 0.237 | 0.299 | 0.125 | 0.093 | 0.126 | 0.021 | 0.011 | 0.021 | 0.043 | 0.047 | 0.042 |
| T18 | 0.268 | 0.313 | 0.268 | 0.148 | 0.164 | 0.149 | 0.013 | 0.015 | 0.013 | 0.122 | 0.118 | 0.123 |
| T19 | 0.250 | 0.210 | 0.224 | 0.151 | 0.123 | 0.148 | 0.015 | 0.010 | 0.015 | 0.084 | 0.077 | 0.068 |
| T20 | 0.307 | 0.247 | 0.303 | 0.237 | 0.150 | 0.237 | 0.010 | 0.004 | 0.010 | 0.069 | 0.079 | 0.065 |
| T21 | 0.276 | 0.216 | 0.275 | 0.088 | 0.075 | 0.087 | 0.038 | 0.025 | 0.038 | 0.042 | 0.033 | 0.042 |
| T22 | 0.245 | 0.275 | 0.249 | 0.210 | 0.213 | 0.212 | 0.044 | 0.058 | 0.044 | 0.077 | 0.058 | 0.077 |
| T23 | 0.237 | 0.269 | 0.242 | 0.151 | 0.172 | 0.154 | 0.012 | 0.013 | 0.012 | 0.113 | 0.110 | 0.112 |

Table C.9 The PSDs of three samplers given 2,500 examinees for the psychological scenario.

| Item | ***α*** | | | ***β*** | | | ***g*** | | | ***s*** | | |
| --- | --- | --- | --- | --- | --- | --- | --- | --- | --- | --- | --- | --- |
|  | MMNUTS | Gibbs | NUTS | MMNUTS | Gibbs | NUTS | MMNUTS | Gibbs | NUTS | MMNUTS | Gibbs | NUTS |
| T1 | 0.517 | 0.502 | 0.464 | 0.221 | 0.216 | 0.216 | 0.018 | 0.021 | 0.021 | 0.027 | 0.027 | 0.027 |
| T2 | 0.539 | 0.489 | 0.345 | 0.204 | 0.198 | 0.198 | 0.012 | 0.014 | 0.014 | 0.028 | 0.028 | 0.028 |
| T3 | 0.360 | 0.353 | 0.364 | 0.144 | 0.145 | 0.145 | 0.013 | 0.015 | 0.015 | 0.039 | 0.041 | 0.041 |
| T4 | 0.302 | 0.311 | 0.298 | 0.156 | 0.158 | 0.158 | 0.018 | 0.022 | 0.022 | 0.054 | 0.058 | 0.058 |
| T5 | 0.202 | 0.232 | 0.203 | 0.129 | 0.135 | 0.135 | 0.022 | 0.027 | 0.027 | 0.064 | 0.077 | 0.077 |
| T6 | 0.245 | 0.274 | 0.243 | 0.146 | 0.147 | 0.147 | 0.045 | 0.055 | 0.055 | 0.028 | 0.031 | 0.031 |
| T7 | 0.327 | 0.389 | 0.333 | 0.188 | 0.194 | 0.194 | 0.041 | 0.051 | 0.051 | 0.033 | 0.037 | 0.037 |
| T8 | 0.258 | 0.289 | 0.258 | 0.138 | 0.143 | 0.143 | 0.024 | 0.029 | 0.029 | 0.049 | 0.056 | 0.056 |
| T9 | 0.277 | 0.298 | 0.202 | 0.223 | 0.226 | 0.226 | 0.023 | 0.029 | 0.029 | 0.126 | 0.147 | 0.147 |
| T10 | 0.185 | 0.188 | 0.186 | 0.089 | 0.090 | 0.090 | 0.015 | 0.017 | 0.017 | 0.032 | 0.038 | 0.038 |
| T11 | 0.214 | 0.224 | 0.214 | 0.097 | 0.098 | 0.098 | 0.011 | 0.013 | 0.013 | 0.042 | 0.049 | 0.049 |
| T12 | 0.443 | 0.439 | 0.428 | 0.241 | 0.229 | 0.229 | 0.030 | 0.037 | 0.037 | 0.028 | 0.028 | 0.028 |
| T13 | 0.412 | 0.403 | 0.150 | 0.236 | 0.244 | 0.244 | 0.013 | 0.015 | 0.015 | 0.098 | 0.111 | 0.111 |
| T14 | 0.219 | 0.240 | 0.218 | 0.150 | 0.154 | 0.154 | 0.019 | 0.023 | 0.023 | 0.091 | 0.107 | 0.107 |
| T15 | 0.294 | 0.310 | 0.288 | 0.161 | 0.164 | 0.164 | 0.017 | 0.021 | 0.021 | 0.065 | 0.073 | 0.073 |
| T16 | 0.219 | 0.244 | 0.186 | 0.170 | 0.175 | 0.175 | 0.015 | 0.019 | 0.019 | 0.119 | 0.137 | 0.137 |
| T17 | 0.211 | 0.217 | 0.211 | 0.097 | 0.098 | 0.098 | 0.010 | 0.011 | 0.011 | 0.048 | 0.056 | 0.056 |
| T18 | 0.233 | 0.280 | 0.236 | 0.181 | 0.195 | 0.195 | 0.015 | 0.017 | 0.017 | 0.081 | 0.098 | 0.098 |
| T19 | 0.201 | 0.220 | 0.182 | 0.148 | 0.150 | 0.150 | 0.009 | 0.011 | 0.011 | 0.109 | 0.127 | 0.127 |
| T20 | 0.224 | 0.223 | 0.215 | 0.152 | 0.137 | 0.137 | 0.003 | 0.003 | 0.003 | 0.106 | 0.124 | 0.124 |
| T21 | 0.169 | 0.179 | 0.169 | 0.093 | 0.092 | 0.092 | 0.018 | 0.021 | 0.021 | 0.033 | 0.039 | 0.039 |
| T22 | 0.235 | 0.275 | 0.237 | 0.204 | 0.210 | 0.210 | 0.059 | 0.070 | 0.070 | 0.049 | 0.059 | 0.059 |
| T23 | 0.221 | 0.269 | 0.215 | 0.178 | 0.190 | 0.190 | 0.013 | 0.015 | 0.015 | 0.097 | 0.117 | 0.117 |

Table C.10 The bias of three samplers given 5,000 examinees for the psychological scenario.

| Item | ***α*** | | | ***β*** | | | ***g*** | | | ***s*** | | |
| --- | --- | --- | --- | --- | --- | --- | --- | --- | --- | --- | --- | --- |
|  | MMNUTS | Gibbs | NUTS | MMNUTS | Gibbs | NUTS | MMNUTS | Gibbs | NUTS | MMNUTS | Gibbs | NUTS |
| T1 | 0.074 | -0.008 | 0.047 | 0.092 | 0.068 | 0.068 | 0.010 | 0.000 | 0.000 | -0.011 | -0.011 | -0.011 |
| T2 | 0.281 | 0.169 | -0.124 | 0.072 | 0.036 | 0.036 | 0.013 | 0.004 | 0.004 | -0.003 | -0.003 | -0.003 |
| T3 | 0.185 | 0.105 | 0.180 | 0.059 | 0.020 | 0.020 | 0.014 | 0.004 | 0.004 | 0.000 | -0.002 | -0.002 |
| T4 | 0.174 | 0.098 | 0.180 | 0.089 | 0.058 | 0.058 | 0.024 | 0.010 | 0.010 | -0.005 | -0.012 | -0.012 |
| T5 | 0.136 | 0.070 | 0.137 | 0.059 | 0.041 | 0.041 | 0.022 | 0.008 | 0.008 | 0.014 | -0.010 | -0.010 |
| T6 | 0.140 | 0.125 | 0.143 | 0.030 | 0.025 | 0.025 | 0.040 | 0.029 | 0.029 | 0.005 | 0.002 | 0.002 |
| T7 | 0.078 | 0.040 | 0.081 | 0.066 | 0.049 | 0.049 | 0.019 | 0.003 | 0.003 | -0.008 | -0.010 | -0.010 |
| T8 | 0.164 | 0.128 | 0.162 | 0.064 | 0.039 | 0.039 | 0.022 | 0.011 | 0.011 | 0.001 | -0.003 | -0.003 |
| T9 | 0.050 | -0.035 | -0.004 | 0.207 | 0.196 | 0.196 | 0.017 | -0.001 | -0.001 | -0.083 | -0.128 | -0.128 |
| T10 | 0.203 | 0.109 | 0.198 | 0.061 | 0.034 | 0.034 | 0.025 | 0.011 | 0.011 | 0.016 | 0.002 | 0.002 |
| T11 | 0.146 | 0.068 | 0.142 | 0.074 | 0.044 | 0.044 | 0.012 | 0.003 | 0.003 | 0.005 | -0.008 | -0.008 |
| T12 | 0.101 | 0.067 | 0.108 | 0.055 | 0.037 | 0.037 | 0.012 | 0.000 | 0.000 | -0.008 | -0.009 | -0.009 |
| T13 | 0.149 | 0.048 | -0.303 | 0.152 | 0.105 | 0.105 | 0.013 | 0.002 | 0.002 | -0.030 | -0.044 | -0.044 |
| T14 | 0.131 | 0.067 | 0.129 | 0.099 | 0.075 | 0.075 | 0.016 | 0.003 | 0.003 | -0.014 | -0.044 | -0.044 |
| T15 | 0.113 | 0.026 | 0.114 | 0.085 | 0.048 | 0.048 | 0.014 | 0.000 | 0.000 | -0.014 | -0.023 | -0.023 |
| T16 | 0.115 | 0.081 | 0.103 | 0.096 | 0.070 | 0.070 | 0.009 | 0.002 | 0.002 | -0.023 | -0.047 | -0.047 |
| T17 | 0.161 | 0.076 | 0.159 | 0.086 | 0.049 | 0.049 | 0.016 | 0.007 | 0.007 | 0.007 | -0.007 | -0.007 |
| T18 | 0.160 | 0.121 | 0.158 | -0.018 | -0.024 | -0.024 | 0.001 | -0.001 | -0.001 | 0.097 | 0.071 | 0.071 |
| T19 | 0.139 | 0.063 | 0.134 | 0.064 | 0.023 | 0.023 | 0.009 | 0.001 | 0.001 | 0.035 | -0.005 | -0.005 |
| T20 | 0.269 | 0.164 | 0.259 | 0.157 | 0.069 | 0.069 | 0.007 | 0.002 | 0.002 | 0.068 | 0.039 | 0.039 |
| T21 | 0.152 | 0.060 | 0.149 | 0.051 | 0.033 | 0.033 | 0.022 | 0.007 | 0.007 | 0.018 | -0.003 | -0.003 |
| T22 | 0.168 | 0.115 | 0.169 | -0.021 | -0.011 | -0.011 | 0.011 | -0.002 | -0.002 | 0.056 | 0.029 | 0.029 |
| T23 | 0.138 | 0.123 | 0.142 | 0.025 | 0.014 | 0.014 | 0.006 | 0.004 | 0.004 | 0.077 | 0.059 | 0.059 |

Table C.11 The RMSEs of three samplers given 5,000 examinees for the psychological scenario.

| Item | ***α*** | | | ***β*** | | | ***g*** | | | ***s*** | | |
| --- | --- | --- | --- | --- | --- | --- | --- | --- | --- | --- | --- | --- |
|  | MMNUTS | Gibbs | NUTS | MMNUTS | Gibbs | NUTS | MMNUTS | Gibbs | NUTS | MMNUTS | Gibbs | NUTS |
| T1 | 0.288 | 0.287 | 0.283 | 0.148 | 0.130 | 0.154 | 0.013 | 0.013 | 0.013 | 0.020 | 0.021 | 0.020 |
| T2 | 0.466 | 0.421 | 0.249 | 0.162 | 0.146 | 0.254 | 0.014 | 0.011 | 0.011 | 0.019 | 0.020 | 0.034 |
| T3 | 0.284 | 0.244 | 0.276 | 0.125 | 0.111 | 0.124 | 0.015 | 0.010 | 0.015 | 0.030 | 0.032 | 0.030 |
| T4 | 0.228 | 0.186 | 0.232 | 0.126 | 0.105 | 0.126 | 0.025 | 0.016 | 0.026 | 0.034 | 0.039 | 0.034 |
| T5 | 0.170 | 0.144 | 0.171 | 0.098 | 0.094 | 0.099 | 0.025 | 0.020 | 0.026 | 0.040 | 0.049 | 0.039 |
| T6 | 0.212 | 0.232 | 0.209 | 0.112 | 0.112 | 0.113 | 0.047 | 0.046 | 0.048 | 0.020 | 0.021 | 0.020 |
| T7 | 0.144 | 0.142 | 0.141 | 0.133 | 0.123 | 0.131 | 0.025 | 0.023 | 0.025 | 0.022 | 0.023 | 0.021 |
| T8 | 0.214 | 0.216 | 0.214 | 0.108 | 0.096 | 0.109 | 0.026 | 0.021 | 0.026 | 0.032 | 0.035 | 0.032 |
| T9 | 0.169 | 0.211 | 0.119 | 0.256 | 0.240 | 0.266 | 0.022 | 0.019 | 0.021 | 0.111 | 0.151 | 0.115 |
| T10 | 0.243 | 0.183 | 0.236 | 0.087 | 0.073 | 0.087 | 0.026 | 0.014 | 0.026 | 0.030 | 0.032 | 0.030 |
| T11 | 0.198 | 0.166 | 0.193 | 0.091 | 0.070 | 0.091 | 0.013 | 0.009 | 0.013 | 0.030 | 0.037 | 0.030 |
| T12 | 0.270 | 0.325 | 0.276 | 0.176 | 0.171 | 0.176 | 0.018 | 0.021 | 0.019 | 0.021 | 0.022 | 0.020 |
| T13 | 0.269 | 0.268 | 0.309 | 0.228 | 0.208 | 0.438 | 0.014 | 0.008 | 0.008 | 0.075 | 0.091 | 0.161 |
| T14 | 0.197 | 0.210 | 0.195 | 0.149 | 0.136 | 0.147 | 0.020 | 0.017 | 0.020 | 0.062 | 0.085 | 0.060 |
| T15 | 0.198 | 0.165 | 0.199 | 0.136 | 0.119 | 0.139 | 0.016 | 0.010 | 0.016 | 0.046 | 0.053 | 0.046 |
| T16 | 0.171 | 0.218 | 0.145 | 0.141 | 0.126 | 0.141 | 0.013 | 0.014 | 0.013 | 0.080 | 0.103 | 0.071 |
| T17 | 0.192 | 0.145 | 0.190 | 0.107 | 0.083 | 0.107 | 0.017 | 0.010 | 0.017 | 0.033 | 0.040 | 0.033 |
| T18 | 0.198 | 0.174 | 0.193 | 0.141 | 0.146 | 0.141 | 0.012 | 0.013 | 0.012 | 0.104 | 0.084 | 0.103 |
| T19 | 0.166 | 0.124 | 0.156 | 0.101 | 0.083 | 0.101 | 0.010 | 0.007 | 0.010 | 0.068 | 0.068 | 0.063 |
| T20 | 0.303 | 0.212 | 0.285 | 0.171 | 0.093 | 0.169 | 0.007 | 0.002 | 0.007 | 0.089 | 0.076 | 0.083 |
| T21 | 0.169 | 0.105 | 0.167 | 0.077 | 0.070 | 0.078 | 0.023 | 0.012 | 0.023 | 0.029 | 0.030 | 0.029 |
| T22 | 0.218 | 0.212 | 0.221 | 0.158 | 0.176 | 0.162 | 0.041 | 0.049 | 0.042 | 0.060 | 0.042 | 0.061 |
| T23 | 0.179 | 0.213 | 0.187 | 0.102 | 0.109 | 0.104 | 0.009 | 0.009 | 0.009 | 0.087 | 0.086 | 0.089 |

Table C.12 The PSDs of three samplers given 5,000 examinees for the psychological scenario.

| Item | ***α*** | | | ***β*** | | | ***g*** | | | ***s*** | | |
| --- | --- | --- | --- | --- | --- | --- | --- | --- | --- | --- | --- | --- |
|  | MMNUTS | Gibbs | NUTS | MMNUTS | Gibbs | NUTS | MMNUTS | Gibbs | NUTS | MMNUTS | Gibbs | NUTS |
| T1 | 0.375 | 0.372 | 0.363 | 0.150 | 0.145 | 0.145 | 0.015 | 0.019 | 0.019 | 0.019 | 0.019 | 0.019 |
| T2 | 0.338 | 0.383 | 0.251 | 0.147 | 0.138 | 0.138 | 0.010 | 0.011 | 0.011 | 0.019 | 0.019 | 0.019 |
| T3 | 0.246 | 0.254 | 0.248 | 0.102 | 0.101 | 0.101 | 0.010 | 0.012 | 0.012 | 0.027 | 0.029 | 0.029 |
| T4 | 0.200 | 0.206 | 0.203 | 0.109 | 0.107 | 0.107 | 0.015 | 0.017 | 0.017 | 0.038 | 0.042 | 0.042 |
| T5 | 0.149 | 0.164 | 0.151 | 0.094 | 0.096 | 0.096 | 0.019 | 0.023 | 0.023 | 0.054 | 0.066 | 0.066 |
| T6 | 0.180 | 0.206 | 0.183 | 0.105 | 0.105 | 0.105 | 0.039 | 0.047 | 0.047 | 0.022 | 0.023 | 0.023 |
| T7 | 0.209 | 0.229 | 0.210 | 0.125 | 0.123 | 0.123 | 0.034 | 0.043 | 0.043 | 0.025 | 0.026 | 0.026 |
| T8 | 0.188 | 0.207 | 0.190 | 0.096 | 0.097 | 0.097 | 0.020 | 0.024 | 0.024 | 0.035 | 0.038 | 0.038 |
| T9 | 0.217 | 0.220 | 0.173 | 0.174 | 0.176 | 0.176 | 0.021 | 0.026 | 0.026 | 0.109 | 0.131 | 0.131 |
| T10 | 0.129 | 0.128 | 0.130 | 0.062 | 0.062 | 0.062 | 0.011 | 0.013 | 0.013 | 0.025 | 0.028 | 0.028 |
| T11 | 0.152 | 0.157 | 0.151 | 0.069 | 0.068 | 0.068 | 0.009 | 0.010 | 0.010 | 0.033 | 0.038 | 0.038 |
| T12 | 0.309 | 0.338 | 0.319 | 0.162 | 0.161 | 0.161 | 0.025 | 0.032 | 0.032 | 0.019 | 0.020 | 0.020 |
| T13 | 0.285 | 0.293 | 0.105 | 0.157 | 0.168 | 0.168 | 0.010 | 0.012 | 0.012 | 0.054 | 0.068 | 0.068 |
| T14 | 0.166 | 0.176 | 0.163 | 0.110 | 0.112 | 0.112 | 0.015 | 0.019 | 0.019 | 0.078 | 0.091 | 0.091 |
| T15 | 0.202 | 0.201 | 0.202 | 0.112 | 0.114 | 0.114 | 0.015 | 0.017 | 0.017 | 0.045 | 0.050 | 0.050 |
| T16 | 0.173 | 0.187 | 0.157 | 0.124 | 0.129 | 0.129 | 0.012 | 0.015 | 0.015 | 0.102 | 0.117 | 0.117 |
| T17 | 0.145 | 0.146 | 0.144 | 0.069 | 0.069 | 0.069 | 0.008 | 0.009 | 0.009 | 0.038 | 0.043 | 0.043 |
| T18 | 0.164 | 0.172 | 0.165 | 0.128 | 0.125 | 0.125 | 0.012 | 0.013 | 0.013 | 0.071 | 0.083 | 0.083 |
| T19 | 0.140 | 0.145 | 0.134 | 0.104 | 0.105 | 0.105 | 0.007 | 0.009 | 0.009 | 0.097 | 0.108 | 0.108 |
| T20 | 0.179 | 0.172 | 0.176 | 0.103 | 0.095 | 0.095 | 0.002 | 0.002 | 0.002 | 0.096 | 0.111 | 0.111 |
| T21 | 0.108 | 0.109 | 0.108 | 0.063 | 0.062 | 0.062 | 0.014 | 0.016 | 0.016 | 0.026 | 0.030 | 0.030 |
| T22 | 0.179 | 0.187 | 0.179 | 0.155 | 0.159 | 0.159 | 0.046 | 0.054 | 0.054 | 0.041 | 0.048 | 0.048 |
| T23 | 0.146 | 0.169 | 0.150 | 0.126 | 0.132 | 0.132 | 0.010 | 0.011 | 0.011 | 0.079 | 0.095 | 0.095 |

# Appendix E: The items of tobacco and alcohol use in the empirical example.

The datasets including the responses of 11,154 individuals to 12 Tobacco and Alcohol Use (TAU) items from the 2009-2010 Health Behavior in School-Aged Children (HBSC) research (Iannotti, 2013; doi: 10.3886/ICPSR34792.v1) were analyzed.

The dataset can be also obtained from <https://www.icpsr.umich.edu/web/NAHDAP/studies/34792/publications>

The 12 items are as follows:

**1. Have you ever smoked tobacco?**  Yes No

**2. How often do you smoke tobacco at present?**

Every day

At least once a week, but not every day

Less than once a week

I do not smoke

**3. At present, how often do you drink beer?**

Never Rarely Every month Every week Every day

**4. At present, how often do you drink wine?**

Never Rarely Every month Every week Every day

**5. At present, how often do you drink liquor/spirits?**

Never Rarely Every month Every week Every day

**6. At present, how often do you drink Pre-mixed drinks (for example, Smirnoff Ice, Bacardi** Breezer, Mike’s Hard Lemonade)?

Never Rarely Every month Every week Every day

**7. At present, how often do you drink any other drink that contains alcohol?**

Never Rarely Every month Every week Every day

**8. On how many occasions (if any) have you smoked cigarettes in the last 30 days?**

Never Once or twice 3-5 times 6-9 times 10-19 times 20-39 times 40 times or more

**9. On how many occasions (if any) have you drunk alcohol in the last 30 days?**

Never Once or twice 3-5 times 6-9 times 10-19 times 20-39 times 40 times or more

**10. On how many occasions (if any) have you been drunk in the last 30 days?**

Never Once or twice 3-5 times 6-9 times 10-19 times 20-39 times 40 times or more

**11. How frequently have you smoked cigarettes during the LAST 30 DAYS?**

Not at all

Less than 1 cigarette per week

Less than 1 cigarette per day

1-5 cigarettes per day

6-10 cigarettes per day

11-20 cigarettes per day

More than 20 cigarettes per day

**12. Have you ever had so much alcohol that you were really drunk?**

No, never Yes, once Yes, 2-3 times Yes, 4-10 times Yes, more than 10 times

Béguin, A. A., & Glas, C. A. W. (2001). MCMC estimation and some model-fit analysis of multidimensional IRT models [journal article]. *Psychometrika*, *66*(4), 541-561. <https://doi.org/10.1007/bf02296195>

Culpepper, S. A. (2015). Revisiting the 4-parameter item response model: Bayesian estimation and application. *Psychometrika*, *81*(4), 1142-1163. <https://doi.org/10.1007/s11336-015-9477-6>

Guo, S., Chen, Y., Zheng, C., & Li, G. (2023). Mixture-modelling-based Bayesian MH-RM algorithm for the multidimensional 4PLM. *British Journal of Mathematical and Statistical Psychology*, *76*(3), 585-604. <https://doi.org/10.1111/bmsp.12300>

Guo, S., Wu, T., Zheng, C., & Chen, Y. (2021). Bayesian modal estimation for the one parameter logistic ability-based guessing (1PL-AG) model. *Applied Psychological Measurement*, *45*(3), 195–213. <https://doi.org/10.1177/0146621621990761>

Zheng, C., Guo, S., & Kern, J. L. (2021). Fast Bayesian estimation for the four-parameter logistic model (4PLM). *Sage Open*, *11*(4), 1-13. <https://doi.org/10.1177/21582440211052556>
